# Describing, analysing and understanding the effects of the introduction of HIV self-testing in West Africa through the ATLAS programme in Côte d’Ivoire, Mali and Senegal

**DOI:** 10.1186/s12889-021-10212-1

**Published:** 2021-01-21

**Authors:** Nicolas Rouveau, Odette Ky-Zerbo, Sokhna Boye, Arlette Simo Fotso, Marc d’Elbée, Mathieu Maheu-Giroux, Romain Silhol, Arsène Kra Kouassi, Anthony Vautier, Clémence Doumenc-Aïdara, Guillaume Breton, Abdelaye Keita, Eboi Ehui, Cheikh Tidiane Ndour, Marie-Claude Boilly, Fern Terris-Prestholt, Dolorès Pourette, Alice Desclaux, Joseph Larmarange, for the ATLAS Team, Marie-Claude Boily, Alice Desclaux, J. oseph Larmarange, Dolorès Pourette, Fern Terris-Prestholt, Abdelaye Keita, Arlette Simo Fotso, Arsène Kouassi Kra, Anne Bekelynck, Guillaume Breton, Marc d’Elbée, Desgree du Lou Annabel, Elvis Georges Amani, Jean Kévin, Ky-zerbo Odette, Kéba Badiane, Maheu-Giroux Mathieu, Moh Raoul, Mosso Rosine, Métogara Mohamed Traore, Paltiel David, Eboi Ehui, Silhol Romain, Rouveau Nicolas, Sokhna Boye, Clémence Doumenc-Aïdara, Sanata Diallo, Odé Kanku Kabemba, Olivier Geoffroy, Vautier Anthony, Alain-Michel Kpolo, Annie Diokouri, Armand Abokon, Blaise Kouame, Camille Anoma, Venance Kouakou, Odette Koffi, Josiane Tety, Yacouba Traore, Abdoulaye S. Anogo, Daouda Diakite, Djelika Berthé, Camara Adam Yattassaye, Dembele Bintou Keita, Dramane Koné, Jules Bagendabanga, Aminata Saran Keita, Septime Hessou, Telly Nouhoum, Fadiala Sidibé, Kanoute Abdul Karim, Madani Tall, Mahamadou Diakite, Maiga Almoustapha, Mariam Koné, Minta Daouda, Saidou Kanambaye, Youssouf Diallo, Alassane Moussa Niang, Fatou Fall, Idrissa Bâ, N Dèye Fatou N Gom Guèye, Oumar Samba, Papa Amadou Niang, Safiatou Thiam, Nguissali M. E. Turpin, Sidy Mokhtar NDiaye, Brou Alexis Kouadio, Cheick Sidi Camara, Sarrassat Sophie, Seydou Bouaré, Souleymane Sow, Ndour Cheikh Tidiane

**Affiliations:** 1 Ceped (Centre Population & Développement UMR 196), IRD, Université de Paris, Inserm, Paris, France; 2 Institut de Recherche pour le Développement, Transvihmi (UMI 233 IRD, 1175 INSERM, Montpellier University), Montpellier, France; 3 Department of Global Health and Development, Faculty of Public Health and Policy, London School of Hygiene and Tropical Medicine, London, UK; 4 Department of Epidemiology, Biostatistics, and Occupational Health, School of Population and Global Health, McGill University, Montréal, QC H3A 1A2 Canada; 5 Analysis Department of Infectious Disease Epidemiology, Medical Research Council Centre for Global Infectious Disease, Imperial College London, London, UK; 6 Solthis, Dakar, Sénégal; 7 Solthis, Paris, France; 8 Institut National de Recherche en Santé Publique (INRSP), Bamako, Mali; 9 Programme National de Lutte contre le Sida, Abidjan, Côte d’Ivoire; 10 Division de Lutte contre le Sida et les IST, Ministère de la Santé et de l’Action sociale, Dakar, Sénégal; 11 CRCF, Dakar, Sénégal

**Keywords:** HIV/AIDS, HIV self-testing, West Africa, Senegal, Côte d’Ivoire, Mali

## Abstract

**Background:**

The ATLAS programme aims to promote and implement HIV self-testing (HIVST) in three West African countries: Côte d’Ivoire, Mali, and Senegal. During 2019–2021, in close collaboration with the national AIDS implementing partners and communities, ATLAS plans to distribute 500,000 HIVST kits through eight delivery channels, combining facility-based, community-based strategies, primary and secondary distribution of HIVST.

Considering the characteristics of West African HIV epidemics, the targets of the ATLAS programme are hard-to-reach populations: key populations (female sex workers, men who have sex with men, and drug users), their clients or sexual partners, partners of people living with HIV and patients diagnosed with sexually transmitted infections and their partners.

The ATLAS programme includes research support implementation to generate evidence for HIVST scale-up in West Africa.

The main objective is to describe, analyse and understand the social, health, epidemiological effects and cost-effectiveness of HIVST introduction in Côte d’Ivoire, Mali and Senegal to improve the overall HIV testing strategy (accessibility, efficacy, ethics).

**Methods:**

ATLAS research is organised into five multidisciplinary workpackages (WPs):

- *Key Populations WP:* qualitative surveys (individual in-depth interviews, focus group discussions) conducted with key actors, key populations, and HIVST users.
- *Index testing WP:* ethnographic observation of three HIV care services introducing HIVST for partner testing.
- *Coupons survey WP:* an anonymous telephone survey of HIVST users.
- *Cost study WP:* incremental economic cost analysis of each delivery model using a top-down costing with programmatic data, complemented by a bottom-up costing of a representative sample of HIVST distribution sites, and a time-motion study for health professionals providing HIVST.
- *Modelling WP:* Adaptation, parameterisation and calibration of a dynamic compartmental model that considers the varied populations targeted by the ATLAS programme and the different testing modalities and strategies.

**Discussion:**

ATLAS is the first comprehensive study on HIV self-testing in West Africa. The ATLAS programme focuses particularly on the secondary distribution of HIVST. This protocol was approved by three national ethic committees and the WHO’s Ethical Research Committee.

**Supplementary Information:**

The online version contains supplementary material available at 10.1186/s12889-021-10212-1.

## Background

In West Africa, HIV prevalence ranges from 0.3 to 3.4% in the general adult population [1]. These epidemics are usually classified as “generalised” (above 1%). Despite this, key populations (female sex workers (FSW), men who have sex with men (MSM), people who use injectable drugs (PWuID)) and vulnerable populations (depending on the country: men in uniform, mobile workers, clients of FSW, etc.) are important to local HIV transmission dynamics [2].

In 2019, only 68% of people living with HIV (PLHIV) in West Africa were aware of their HIV status, 58% were on antiretroviral (ARV) therapy and 45% had an undetectable viral load [1], all of which are far from the levels required to achieve epidemic control. The first step of the HIV care cascade, HIV testing, is also the most important gap to fill to attain the 90–90-90 UNAIDS targets [3]. The WHO/UNAIDS meeting held in Dakar in November 2017 highlighted the urgent need to accelerate progress on HIV testing in West Africa and to adopt innovative and tailored testing approaches to reach those who have not yet been tested [4].

Over the past decade, considering the concentrated aspect of local epidemics, national AIDS programmes have increasingly developed actions specifically targeting key populations who are particularly vulnerable to HIV acquisition and transmission. Community-based activities have thus improved access to HIV testing among those reached by peer educators, FSW and MSM in particular, while activities targeting PWuID were less common.

Despite these initiatives, key populations remain difficult to reach, particularly hidden MSM and occasional FSW [5]. While HIV stigma and distance from services are common barriers to HIV testing for the entire population in West Africa, key populations exhibit lower HIV testing rates and face specific additional barriers due to socially stigmatised and even illegal practices [6–9].

Epidemic dynamics are complex and understanding heterogeneity in the risk of HIV acquisition and transmission is crucial for maximising the impact of the HIV response [10]. Approximately 30% of new infections are estimated to occur among people who engage in low-risk behaviours but have partners with high-risk behaviours, such as unprotected sex with a partner whose HIV status is unknown or multiple unprotected sexual partnerships [11]. According to two modelling studies, 44% of new infections arising in Côte d’Ivoire from 2005 to 2015 occurred in partnerships between clients and their non-FSW female partners [12], while over the same period, 38% of new HIV infections occurring in Dakar and Senegal were within non-commercial partnerships between FSW and clients [13].

### HIV self-testing: an innovative and powerful tool for testing with remaining research gaps

HIV self-testing (HIVST) is a process in which the user takes a sample (oral fluid or blood), performs the HIV test, and then interprets the result alone, often in a private setting (WHO 2016).

Several studies have shown that, for many users, HIVST promotes discretion and autonomy, HIV test uptake by first-time testers and is associated with increased testing frequency [14–17]. HIVST has high acceptability, particularly among key populations and those who do not test regularly [18–21].

Distribution strategies for HIVST can be based on a primary distribution where the HIVST kit is given directly to the person who will perform the test [20, 22] or on a secondary distribution where the HIVST is given to a relay person who will redistribute it to one or many of his/her contacts. Secondary distribution makes it possible to reach hidden key populations, such as clients of FSW [23, 24], partners of MSM [25], or index partners of pregnant women [26–28] who are not routinely tested. The ability of primary contacts to distribute HIVST kits to their contacts is not guaranteed, as secondary distribution may be limited due to specific barriers and may be accompanied by social consequences, such as acts of violence against FSW by their clients [22, 23].

In sub-Saharan Africa, partners of PLHIV are among the priority target populations for testing because they are at particular risk for HIV infection. Indeed, some studies estimate that up to two-thirds of new infections occur in heterosexual couples [29]. In many couples, one or both partners do not know their HIV status [30]. Difficulties in disclosing one’s status due to HIV-related stigma, leading to low rates of partner sharing, have been reported [31–35]. HIVST is a complementary tool to other HIV testing support activities for partners of PLHIV. HIVST can be a way to initiate a dialogue on HIV between partners when a partner is not informed. This disclosure can have consequences for the couple (separation, violence, dialogue, support) [36, 37] and also for the partner’s willingness to undergo HIVST. In all these issues, the gender dimension is an important element. Indeed, men and women do not manage disclosure of HIV status similarly, nor do they manage the relationship to HIV test results in the same way [38].

Sexually transmitted infections (STI) counselling provides an opportunity for HIV testing for both patients and their partners [39]. People infected with sexually transmitted infections (STIs) are also at higher risk for HIV infection [40]. It has been shown that the use of STI counselling among individuals who thought they were at risk of HIV infection was higher than that among those who did not think they were at risk. Furthermore, although HIV testing at STI consultations is recommended, the percentage of testing initiated in this context remains low. A recent study in Côte d’Ivoire found that only 28% of patients who had an STI medical consultation were offered HIV testing [41].

Only a few pilot experiments in HIV self-testing have been implemented in West Africa. There is a strong demand for the implementation of new innovative prevention and testing tools, such as HIVST, in the region [42, 43]. One study in Senegal showed that HIVST represents an approach that reached first-time testers and those who had not been tested recently [44]. The introduction of HIV self-testing in West Africa may help to remove some of the barriers faced by key and vulnerable populations and improve their access to HIV testing, as observed in other parts of Africa [25, 45].

### The ATLAS programme

Following the WHO recommendations and the experience gained in East and Southern Africa through the STAR programme [46], Unitaid and other countries wished to promote and implement HIV self-testing in West Africa through funding the ATLAS programme in Côte d’Ivoire, Mali and Senegal. During 2019–2021, ATLAS plans to dispense more than 500,000 HIV self-test kits. Only oral HIVST OraQuick HIV Self-Test® (OraSure Technologies, LLC Bethlehem) will be used as it is pre-qualified by WHO and has been validated by the three countries of intervention.

In each country, the provision of HIVST is complementary and integrated into existing HIV testing strategies and will be performed by field workers already engaged in testing activities financed by the Global Fund or PEPFAR. Different delivery channels and target populations for each country have been developed in collaboration with the countries’ stakeholders (national AIDS programmes/councils, international institutions, including WHO, international and national NGOs involved in local HIV programmes, civil society, communities).

**Fig. 1:**
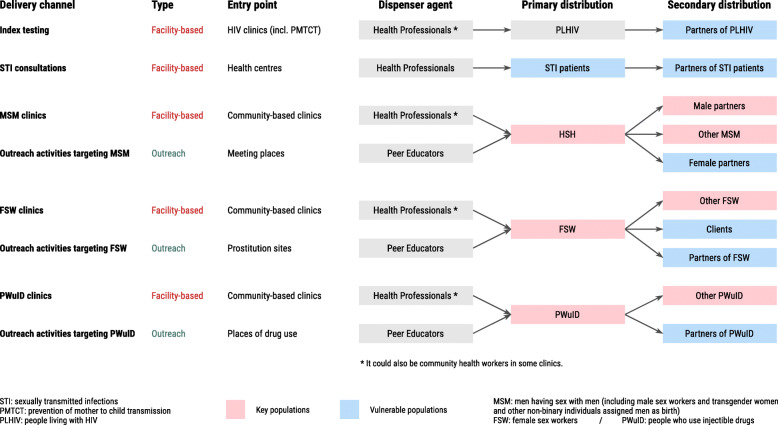
ATLAS delivery channels

Eight delivery channels have been selected for the ATLAS programme after discussion with various stakeholders (Fig. 1). Five delivery channels adopt a facility-based strategy (delivery of HIVST in a health facility), and three delivery channels adopt a community-based strategy through field activities.

Due to the West African epidemiological and social context, the ATLAS programme plans to promote secondary distribution in each of the eight delivery channels to reach beyond those reached through primary distribution by health care workers and peer educators.

### Research needs

Implementation of HIVST in West Africa raises specific questions related to the cultural, social, economic, and epidemiological contexts of this region. Furthermore, questions remain regarding specific delivery channels and secondary distribution in particular.

The social dimensions of HIV testing in general, and of HIVST in particular, play an important role and impact the acceptability and uptake of HIVST in different populations and contexts [47]. Regardless of the perceptions and attitudes of populations and health workers, the quality and organisation of health services is an essential factor that can promote or limit access to testing. More generally, documentation of the factors facilitating or limiting HIVST is a way to support the implementation of tailored activities.

Because of the large proportion of HIVST secondary distribution, routinely collected programmatic data will be insufficient to describe the sociodemographic profiles of HIVST users, as well as their trajectories of care following a reactive HIVST. In addition to its health impact, HIVST could have other social consequences, harmful or beneficial, not foreseen by the health system. For example, some people could be coerced by their partner to be tested, and a reactive result could be a source of violence. Despite challenges, documenting the profile of HIVST users and the social and health consequences of HIVST is essential.

With HIVST being integrated with other conventional HIV testing strategies, it is important to evaluate the routine incremental costs of adding HIVST to existing services and its associated epidemiological impacts for each delivery channel. These data are essential for national programmes and technical and financial partners to prepare for scaling up by comparing different possible scenarios of distribution channels, population and estimating cost-effectiveness and budgetary impacts.

### Scientific objectives

The primary objective of the research component embedded in the ATLAS programme is to describe, analyse and understand the social, health, epidemiological and economic effects of the introduction of HIV self-testing in Côte d’Ivoire, Mali and Senegal to improve the availability of testing (accessibility, efficacy and ethics).

This primary objective can be subdivided into six secondary objectives:

- ➔ Identify the social, cultural and organisational factors facilitating and limiting the primary and secondary distribution of HIV self-tests and their use/appropriation by the different actors concerned (programme or project manager and NGO representative, delivery agents, primary contacts, secondary contacts).
- ➔ Establish the socio-behavioural profile and HIV testing history of HIV self-test users and their care history in the event of a reactive self-test.
- ➔ Analyse the positive and negative social and health consequences of the introduction of HIV self-testing for individuals, communities and the health system.
- ➔ Estimate the incremental costs of dispensing HIV self-tests per delivery channel.
- ➔ Model the epidemiological impacts of the ATLAS programme and different scaling scenarios on epidemic dynamics.
- ➔ Estimate the medium- and long-term cost-effectiveness and budgetary impact of different strategies for scaling-up.

## Methods and analysis

This protocol is based on version 2.1 of the ATLAS research protocol. In case of future updates, the latest version of the protocol will be available on https://atlas.solthis.org/recherche/.

The ATLAS research component brings together multidisciplinary researchers from different institutions in partnership with the different operational teams of the programme.

Research activities embedded within the ATLAS programme are organised into 5 work packages: (i) a qualitative survey on HIVST focused on key populations and based on qualitative individual and group interviews with key implementers, members of key population communities and HIVST users; (ii) an ethnography on the integration of HIVST for screening PLHIV’s partners in three HIV care clinics; (iii) an anonymous telephone survey of HIVST users recruited through an invitation on HIVST kits to call a toll-free number; (iv) an economic survey of HIVST incremental costs with cost collections from a sample of HIVST dispensing sites and a time and motion study; (v) an epidemiological modelling (dynamic compartmental model) of the three countries and of the health and economic impacts of different scaling scenarios.

The five work packages (WPs) with the six secondary objectives (SO) and specific objectives are summarised in Tables 1 and 2.

**Table 1:** Work package and secondary objectives

Work package	Key populations	Indextesting	Couponssurvey	Cost study	Modelling
**Delivery channels**	FSW, MSM, PWuID	Index testing (partners ofPLHIV)	All	All	All
**Study populations**	Key actors, Community members, HIVST users	PLHIV andpartners, Health workers	HIVST users	Dispensing sites, Dispensing agents	–
**SO1** Identify the social, cultural and organisational factorsfacilitating and limiting the primary and secondary distributionof HIVST kits and their use by the different actors involved.	✓	✓			
**SO2** Define the socio-behavioural profile and HIV testinghistory of HIVST users and their care pathway in the eventof a reactive test.	✓		✓		
**SO3** Analyse the positive and negative social and healthconsequences of the introduction of HIVST for individuals,communities and the health system.	✓	✓	✓		
**SO4** Estimate the incremental costs of dispensing HIVSTkits per dispensing channel.				✓	
**SO5** Model the epidemiological impacts of the ATLASprogramme and different scale-up scenarios on epidemicdynamics.			✓		✓
**SO6** Estimate the cost-effectiveness and the medium-and long-term budgetary impact of different scaling strategies.				✓	✓

**Table 2:** Work packages and specific objectives

Work package	Key populations	Index testing	Coupons survey	Cost study	Modelling
**Specific objectives**	- Identify factors favouring and limiting the introduction of HIVST in the health care system (public and community) and primary and secondary distribution of HIVST kits to key populations. - Analyse perceptions, attitudes, ownership, experience and living with HIVST - Analyse the social effects of HIV prevention. - At the individual, collective and care system levels, in each of the three countries and among the different key populations, take into account the socio-cultural and political contexts that differ from one country to another and from one key population to another.	- Describe how HIV care services and caregivers integrate the proposal of HIVST for partners of PLHIV. - Study how PLHIV negotiate issues around the HIVST proposal to their partner. - Analyse the perceptions, uses and modalities of use of HIVST by PLHIV partners, as well as the impact of the use of HIVST on the relationship between screening and risk. - Identify the individual, conjugal and social impacts of the HIVST.	- Document the socio-behavioural profile and screening history of HIVST users. - Identify the care trajectories of these HIVST users following a reactive or indeterminate self-test. - Provide an empirical estimation of certain parameters used by the Modelling WP.	- Estimate the incremental costs of providing HIV kits by country and by delivery channel based on observed programme costs. - Compare the costs of HIV testing and counselling with other HIV testing and counselling approaches for the same populations in ATLAS countries. - Model the costs of scaling up (regionally and/or nationally) a combination of medium (3–5 years) and long-term HIV HIVST delivery models for budget planning purposes. - Compare the costs with the expected epidemiological impacts (Modelling WP) to estimate the cost-effectiveness of these scale-up scenarios.	- Identify those most likely to acquire and transmit HIV and identify delays in testing and diagnosis. - Estimate the population-level impact of the introduction of HIVST in the three ATLAS countries, (i) at the scale achieved by the ATLAS programme and (ii) within the framework of possible scenarios for scaling up. - Estimate the cost-effectiveness of these scale-up scenarios and conduct a sensitivity analysis to determine the conditions and factors that influence cost-effectiveness.

### Key populations WP

The specific objectives of this WP are to identify factors that promote and limit the integration of HIV self-testing into the health care system and the primary and secondary distribution of HIV self-tests in key populations (FSW, MSM and PWuID); to analyse perceptions, attitudes, ownership, and experience of HIV self-testing; and to analyse the social effects of HIV self-testing at the individual, collective and health system level.

Qualitative surveys will be conducted (individual in-depth interviews and focus group discussions) in the three countries with (i) key actors in testing programmes targeting key populations (FSW, MSM, PWuID); (ii) members of the three key population communities and (iii) HIV self-test users recruited either by peer educators or through the Coupons survey. The different sub-studies are described in Table 3 and will be conducted sequentially. They will be complemented by document review (press articles, HIVST promotional documents).

**Table 3:** Key populations WP

Sub-study	Aims	Population	Methods	Themes explored
Sub-study 1: Factors related to the introduction of HIVST in the health care system	To assess the perception of HIVST as a factor favouring and limiting the deployment of HIVST among key populations.	Public health stakeholders, association and representatives of key populations	15–20 in-depth interviews will be conducted in each country.	Difficulties, opportunities and obstacles to the introduction of HIVST and its support system in the country’s associative and health system; Difficulties and obstacles related to secondary distribution; Difficulties and obstacles specific to each population; Perceptions of the support system (advice, green line, tools); Recommended adjustments for key populations.
Sub-study 2: Collective attitudes and perceptions of HIVST	To analyse perceptions, motivations and barriers to use HIVST among key populations	Members of the three key population communities (FSW, MSM, PWuID), whether or not they have used HIVST, identified by peer educators from ATLAS partner community associations	3 focus group discussions (FGDs) will be conducted with members of each key population in each country (i.e., 9 FGDs in Côte d’Ivoire, 6 FGDs in Mali and 9 FGDs in Senegal, i.e., 24 FGDs in total). Each group will be composed of 8 to 10 members of the key population under consideration.	Perceptions of HIVST (information circulating about HIVST in the community, opinions on the advantages/disadvantages of HIVST compared to routine testing, advantages/risks of introducing HIVST in each community); Motivations and barriers for HIVST (self-confidence to carry out the test, testing and testing offer practices, conditions under which HIVST can be accepted/refused, facilitating and limiting factors for the practice of confirmatory testing); Motivations and barriers to secondary distribution; Suggestions for promoting the practice of HIVST for each key population and in each country.
Sub-study 3: Experience of HIVST	To analyse the use of HIVST, the social experience of HIVST users and linkage to care	HIVST users: • identified by peer educators from the community partners of the ATLAS programme (primary distribution) • identified via coupon survey (secondary distribution)	5 in-depth face-to-face interviews will be conducted per key population in each country. 5 additional interviews will be conducted by phone with people recruited through the Coupons survey, who have declared that they have had a reactive self-test and have agreed to be recontacted for an additional qualitative interview during phase 2 of the Coupons survey in each country.	Recourse to HIVST (motivations, circumstances: primary or secondary distribution, perceptions of the process, screening itineraries before HIVST, satisfaction); Social experience (social context of implementation, relations with the applicant, violence or coercion suffered/exercised on the partner/girlfriend, stigmatisation, abuse, changes in terms of prevention strategies and social relations); Difficulties and satisfaction (access to HIVST, implementation, suggestions).
Sub-study 4: Appropriation and integration of HIVST	To explore the level of ownership of HIVST by key populations and key stakeholders and to analyse the integration into the health care system after at least 2 years of implementation of the ATLAS programme	Public health stakeholders, association and representatives of key populations	15–20 interviews will be conducted with the same type of stakeholders as those surveyed in sub-study 1 in each country	Perceptions and attitudes at the end of the intervention (sub-themes similar to survey 1); Integration of the system and impact on the healthcare system; Challenges of providing HIVST to key populations (compared to the general population).

### Index testing WP

The specific objectives of this WP are to describe how HIV care services and healthcare professionals integrate HIV self-testing for sexual partners of PLHIV; to study how PLHIV negotiate issues around the HIV self-testing proposal to their partner(s); to analyse perceptions, uses and modalities of use of HIV self-tests by partners; and to identify individual, marital and social impacts.

This WP will use anthropological methods, consisting primarily of ethnographic observations and individual in-depth interviews.

The study survey will be conducted in all three ATLAS countries, with an ethnographic study in three HIV clinics (one per country) where HIVST is offered as part of the ATLAS programme’s dispensing activities (2.5 months in each clinic). It will include both (i) observations of consultations, medical staff and any other activities organised in connection with the distribution of HIVST; and (ii) in-depth individual interviews with PLHIV followed in the service who were offered an HIVST to distribute to their partners, PLHIV partners who have accepted or refused HIVST and health staff.

At the project end, over 2 weeks, some health workers and patients interviewed during the ethnographic studies will be re-interviewed to evaluate HIVST appropriation by the different actors and the expected and unexpected repercussions and difficulties after several months of implementation.

The content of the interviews and observations will be subject to thematic analysis using the online content analysis software Dedoose. Data analysis will be based on a Grounded Theory approach [48], i.e., a theory developed by induction from a corpus of data. The analytical framework of this WP will be based on a sociological approach to gender [49].

### Coupons survey WP

Specific objectives of the coupons survey are to document the socio-behavioural profile and screening history of HIV self-tests users; to identify the care trajectories of these HIV self-tests users following a reactive or indeterminate self-test; and to provide an empirical estimate of some parameters used by the WP Modelling. The survey was designed to enable data collection from HIVST end-users based on anonymity and voluntary participation through the establishment of an anonymous and free telephone platform in the three countries.

To be eligible for the study, participants must be of legal age to perform an HIV test by him/herself (i.e., 16+ years old in Côte d’Ivoire, 18+ in Mali and 15+ in Senegal) and have performed an HIVST.

The survey will be organised in collection waves with two phases each. A pilot study will be conducted before the implementation of the full survey.

#### Survey phase 1

During phase 1 of each wave, all HIVST kits distributed in the country through ATLAS, regardless of delivery channel, will contain an invitation to participate in the study (the “coupons”). Similarly, anyone calling the national HIV hotline during this period and reporting having performed an HIVST will be invited to participate in the coupons survey by calling the dedicated survey free phone number.

All HIVST kits distributed under the ATLAS programme will also be marked using a coloured sticker with a numbered code to identify the delivery channel and the technical implementation partner. During the telephone interview, the participant will be asked for the colour and number of the sticker to be able to associate each questionnaire with a dispensing channel.

When a person calls one of the study’s toll-free numbers, they will first be presented with the study and then asked to state orally whether they consent to participate (verbal consent, time-stamped). If consent is given, a 15-min questionnaire will be administered. This questionnaire will cover the participant’s socio-demographic characteristics, his or her HIV testing history, sexual practices, knowledge of HIVST, ease of use of the HIVST kit and the result of his/her self-test.

#### Survey phase 2

Phase 1 participants who reported a reactive or indeterminate HIVST result and who agreed to be contacted again will be called back 3 months after completion of the phase 1 questionnaire for the phase 2 questionnaire. This second questionnaire aims to document the participant’s care trajectory and, in particular, whether a confirmatory test has been performed and, if so, whether the person linked to HIV care and initiated antiretroviral treatment.

### Cost study WP

Because HIVST is being added onto existing HIV testing programmes, the specific objectives of the cost WP are to estimate the incremental costs of providing HIVST; to compare the costs of HIVST to other HIV testing approaches; to model medium- and long-term scale-up costs; and to compare costs to expected epidemiological impacts (Modelling WP) to estimate the cost-effectiveness of these scale-up scenarios.

We will conduct an incremental HIVST cost analysis to capture economic costs (i.e., all resources used, including donated goods and services) of the intervention. We will also conduct a full economic cost analysis of a sample of conventional HIV testing sites where HIVST is being introduced. The analysis will follow the recommendations of the Global Health Cost Consortium, which sets standards for health costing studies [50].

Data collection includes (i) a top-down cost approach with programmatic cost data; (ii) a bottom-up cost approach with a representative sample of HIV self-tests distribution sites; and (iii) a time-motion study with a sample of dispensing agents (Table 4).

**Table 4:** Cost study WP

**Top-down approach**	Analysis of programmatic costs (top-down cost) based on Solthis and implementing partners’ financial and activity reports. This analysis will include shared overhead costs of the programme.
**Bottom-up cost approach**	Based on a sample of sites where both HTS and HIVST distribution are ongoing. Dispensing sites will be randomly sampled considering the dispensing strategies implemented in each site (fixed and mobile strategies), the geography of the site (country, region). About 60 sites will be investigated (~ 30 in Côte d’Ivoire, ~ 15 in Mali and ~ 15 in Senegal). The data collected will cover both the costs of dispensing HIV kits and other HIV testing activities conducted on-site. It will include specific costs of the ATLAS programme, as well as costs covered by other donors (e.g., Global Fund, Pepfar) and economic costs not reported in financial reports (e.g., donations of goods and services), as well as allocation factors for the disaggregation of costs by delivery models. The collection will be based on the various reporting documents produced by the structure (financial reports, activity reports, etc.) and on individual interviews conducted with the technical and financial managers of the structure. The interview document with the person in charge on-site will serve as a working basis for the development of a data collection tool by research assistants and will be piloted for adaptation to the different sites in each country.
**Time-motion study**	To complement the bottom-up approach, a time-motion study will be conducted to disaggregate field-based personnel time between HIVST-related activities and other activities to use this information as an allocation factor of personnel costs between HIVST delivery models. The number of providers to be surveyed will be determined based on the preliminary results of the bottom-up approach. Specifically, for each survey day at a given site, the research team will list the providers active on that day and verbally ask each provider if they are willing to participate. Among those who have indicated a willingness to participate in the study, a random draw will be held to select the person(s) to be followed on that day. Written informed consent will be obtained from study participants.

The analysis will take a provider’s perspective. We will estimate programme development, start-up, and implementation costs, capturing both capital costs (infrastructure, equipment, etc.) and recurrent costs (salaries, HIVST kits, etc.). The cost analysis will differentiate between the start-up phase (all costs incurred before the first HIV kits are distributed) and the “implementation” phase (one-year observation period following the beginning of HIVST kit distribution). For each country and delivery channel, the main output will be the total programme costs and average cost per HIVST kit delivered (HIVST cost), and per person tested for HIV/per HIV-positive case identified (HTS cost). Based on the observed costs of early implementation, we will model costs at scale and consider the budgetary impact of alternative HIV testing programme models.

### Modelling WP

The impact of HIVST is not limited to the diagnosis of PLHIV. By identifying more PLHIV unaware of their status (and identifying them more rapidly), there is potential to reduce the average length of time that a person is at risk of transmitting the virus. In this way, HIVST can reduce the morbidity and mortality of newly diagnosed infected people if they are promptly linked to care, as well as reducing onward transmission if they achieve viral load suppression [51].

Such medium- and long-term effects cannot be easily empirically measured, and mathematical models of HIV transmission dynamics offer a robust alternative. For example, different scenarios (with or without the introduction of HIVST, for example) can be compared to estimate incremental gains in new infections prevented, life-years gained, and deaths avoided. Diverse scenarios can be explored, and sensitivity analyses conducted. Epidemic modelling is a valuable tool for answering these types of questions and for providing insights into the possible impacts of different health policies related to HIVST. This tool is also essential for a better understanding of the current epidemic dynamics and improved focus of activities to be performed.

The specific objectives of the Modelling WP are to identify population groups most likely to acquire and transmit HIV and to identify their testing and diagnostic delays; to estimate the population impact of the introduction of HIVST in the three ATLAS countries, at the scale achieved by the ATLAS programme and under possible scale-up scenarios; and to estimate the cost-effectiveness of these scale-up scenarios and conduct sensitivity analysis.

Several existing dynamic models of HIV transmission that have already developed and calibrated to the HIV epidemic in West Africa will be leveraged [12, 13, 52, 53]. These models will be adapted to the idiosyncrasies of HIVST [53], parameterised, and calibrated to detailed epidemiological and intervention data collected as part of the ATLAS programme.

Briefly, the model will represent an open (15–59 years) and growing population stratified by four age groups (15–19, 20–24, 25–49, and 50–59 years). Also, different risk groups and levels of sexual activity will be considered: low- and high-risk women, low- and high-risk men, sex workers, clients of sex workers, men who have sex with men and women, and men who have sex exclusively with men. The model considers the age of sexual debut, as well as the migration and mortality due to HIV or other causes.

**Fig. 2:**
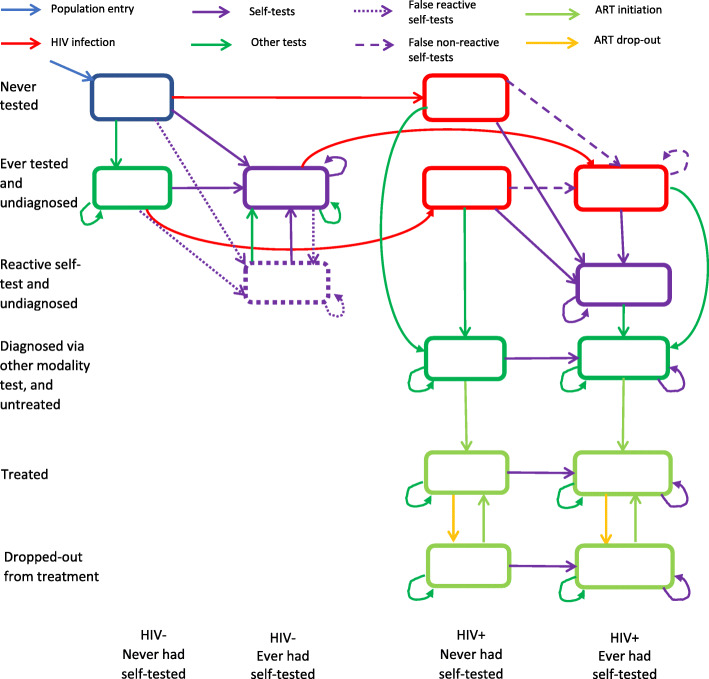
Proposed flowchart describing flows between the main compartments describing testing behaviors and modalities, HIV acquisition, diagnosis, linkage to care, and ART initiation and discontinuation

The model represents natural HIV progression from the initial/acute highly infectious phase to the chronic phase. The model also describes the treatment and care continuum: testing, management, and initiation of antiretroviral therapy. This description of the continuum of care helps to determine the three stages of the UNAIDS 90–90-90 target in each subpopulation group (Fig. 2).

Sexually active individuals at risk may acquire HIV infection at a rate that depends on their annual number of sexual partners and sexual acts, the type of sexual partners and sexual acts (vaginal or anal), the prevalence of HIV among their sexual partners and their stage of infection, and their initiation of treatment, the type of sex, and the rate of condom use.

#### Parameterisation and calibration

The model will have to be parameterised and calibrated for the three ATLAS countries. The model parameters come from four different areas: (i) demographic (age structure, mortality, etc.); (ii) biological (probability of transmission, treatment effectiveness, etc.); (iii) behavioural (number of sexual partners, condom use, etc.) and (iv) national response (testing, antiretroviral coverage, etc.).

The model will then be calibrated within a Bayesian framework to identify the model parameters that best reproduce HIV epidemic trends in the different subgroups, as well as the evolution of the coverage of interventions (e.g., condom use and treatment uptake).

#### Simulations and analyses

Once the three models have been parameterised and calibrated, they will be used to reproduce different hypothetical or observed scenarios:

- The ATLAS programme as implemented
- The screening programme without the implementation of ATLAS
- Various ATLAS scaling scenarios (determined with the economic component)

Last, we compare costs (Cost Study WP) with expected epidemiological impacts to estimate the cost-effectiveness of these scaling scenarios.

### Participants and public involvement

At each stage of the research implementation, communities were involved in the research programme. Stakeholders and local Civil Society Organisations (CSO) participated in programme development workshops which helped to develop the research questions.

Throughout the project, communities, stakeholders and local CSO will be consulted at least once a year through the consortium meetings that bring together all to discuss the progress, results and interpretation of the research. A representative of the Ministry of Health of each country is also associated with the research team which meets annually.

## Discussion

No HIVST kits will be distributed as part of the research activities of this protocol, as all HIVST kits under the ATLAS programme are provided as part of routine testing activities validated by the national AIDS programme of the country involved.

There should be no risk or consequence whatsoever associated with the refusal to participate in one, several or all of the proposed studies. In no way will participation or non-participation in any of the studies conducted impact (positively or negatively) access to HIVST kits. Participation in research will be voluntary.

There is no major individual health risk to participate in these studies. The primary risk is social; if one respondent sees another person participating in this study, he or she may make assumptions about his or her serological status and/or practices, which may be illegal (sex work, sex between men, drug use, etc.) and reveal them to the community. Measures are put in place to avoid this: interviews will be conducted in confidential places, data will be coded and kept in safe places, and records will be destroyed at the end of the project.

A website dedicated to the ATLAS programme (https://atlas.solthis.org) allows participants in the different studies to access the research protocol, the different information records of each study, and the results when they are available.

ATLAS is the first comprehensive study on HIV self-testing in West Africa. The ATLAS programme focuses particularly on the secondary distribution of HIVST. This study will also be the first to analyse the use of HIV self-testing among people who use injectable drugs in Africa. This is a multidisciplinary study that employs both qualitative and quantitative methods.

ATLAS research is an innovative approach to document HIV self-testing use, interfering as less as possible with routine activities and without actively tracking HIVST users to preserve confidentiality and anonymity.

### Ethical approvals

The ATLAS protocol version 2.1 has been reviewed and approved by the following ethical committees:

- Comité National d’Ethique des Sciences de la vie et de la santé de Côte d’Ivoire (ref: 049–19/MSHP/CNESVS-kp, 28th May 2019),
- Comité d’Éthique de la Faculté de Médecine et de Pharmacie de l’université de Bamako Mali (ref: 2019/88/CE/FMPOS, 14th August 2019),
- Comité National d’Ethique pour la Recherche en Santé du Sénégal (ref: SEN19/32, 26th July 2019),
- Ethical Research Committee of the World Health Organisation (ERC 0003181, 7th August 2019),
- LSTHM IRB (ref: 17141, 25th April 2019).

### Consent procedures

Our consent procedures include (i) signed consent form for face-to-face in-depth interviews, focus groups and time and motion study; (ii) specific consent obtained for audio recording interviews; (iii) authorisation of the manager of the dispensing sites and consent of the caregivers for the ethnographic studies and bottom-up cost; and (iv) anonymous time-stamped oral verbal consent for phone surveys.

### Data monitoring plan

A data monitoring plan (DMP) has been developed and specifies the list of data collected in the framework of ATLAS research, their documentation and metadata, ethical and legal aspects, data storage and backup, intellectual property rights, a reminder of ethical aspects, rules for sharing, disseminating and reusing data, and rules for archiving and data preservation. This data management plan is publicly available on the Opidor platform: https://dmp.opidor.fr/plans/3354/export.pdf and is compliant with the European General Data Protection Regulation.

## Supplementary Information

**Additional file 1.**

## Data Availability

The data management plan is publicly available on the Opidor platform: https://dmp.opidor.fr/plans/3354/export.pdf and is compliant with the European General Data Protection Regulation.

## Abbreviation

ARV: Antiretroviral
CSO: Civil Society Organisations
DMP: Data Monitoring Plan
FSW: Female Sex Workers
HIVST: HIV self-testing
HTS: HIV testing services
MSM: Men who have Sex with Men
PLHIV: People Living with HIV
PWuID: People Who use Injectable Drugs
SO: Secondary Objectives
STI: Sexually transmitted infections
WHO: World Health Organisation
WP: Work Package
