# Supplementary material for: Describing, analysing and understanding the effects of the introduction of HIV self-testing in West Africa through the ATLAS programme in Côte d’Ivoire, Mali and Senegal
Source: BMC Public Health. 2021 Jan 21;21:181. doi: 10.1186/s12889-021-10212-1 (PMC7818756; doi:10.1186/s12889-021-10212-1)
Supplement: Supplementary file 1 — Additional file 1. [file 12889_2021_10212_MOESM1_ESM.docx]

**
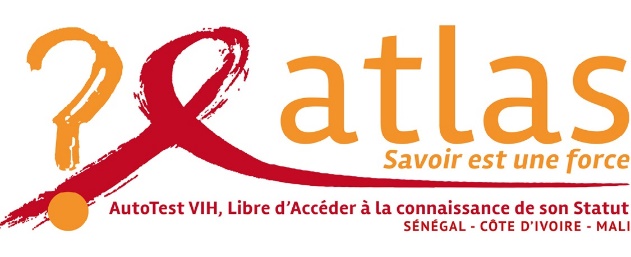
**

**Protocol of the research part of the ATLAS programme**

**Appendices**

List of dispensing………………………………………………………………………. 2

WP Key populations

- WP Key Populations - EIA face-to-face - Information leaflet
- WP Key Populations - Face-to-face EIA - Consent form
- WP Key populations - EIA by phone - Information leaflet
- WP Key populations - Group discussion - Information leaflet
- WP Key Populations - Group discussion - Consent form
- WP Populations Clés - Care Guide

WP Screening of index cases 36

- WP Screening of index cases - Individual interview - Information leaflet
- WP Screening of index cases - Individual interview - Consent form
- WP Screening of index cases - Individual interview - Interview guide
- WP Screening of index cases - Consultation observations - Observation guide

WP Survey Coupons ...49

- WP Survey Coupons - Information leaflet for ATLAS website
- WP Survey Coupons - Long questionnaire phase 1
- WP Survey Coupons - Short questionnaire phase 1
- WP Survey Coupons - Questionnaire phase 2

WP Economic Component ... ... 71

- WP Economy WP - Individual interview - Information leaflet
- WP Economic strand - Individual interview - Consent form
- WP Economy Component - Individual interview - Interview guide
- WP Economic Component - Time-movement study - Information leaflet
- WP Economic Component - Time-movement study - Consent form
- WP Economic Component - Time-movement study - Maintenance guide

Reporting and management of cases of social prejudice and incidents……………………………………………………………………99

Data……………………………………………………………………………………………………………………………102

Summary of the project document validated by Unitaid 111

**List of dispensing sites***

**July ^1,^ 2019 version*

**Ivory Coast :**

**Fixed strategy (32 sites)**

**Region Districts Clinics/Centres**

Gbokle-Nawa-San PedroSan-Pedro Bardot San Pedro Maternal Child Protection

Gbokle-Nawa-San PedroSan-Pedro Grand Bereby Urban Health Centre

Gbokle-Nawa-San PedroSan-Pedro SOGB Medical and Social Centre of Grand-Bereby

Gbokle-Nawa-San PedroSan-Pedro CSU Touih

Gbokle-Nawa-San PedroSan-Pedro Urban Health Training in Zimbabwe

Gbokle-Nawa-San PedroSan-Pedro Gabiadji Urban Health Centre

Gbokle-Nawa-San PedroSan-Pedro Bardo Urban Health Centre

Gbokle-Nawa-San PedroSan-Pedro CMS of SEMPA San-Pedro

Gbokle-Nawa-San PedroSan-Pedro San Pedro Regional Hospital Centre

Gbokle-Nawa-San PedroSan-Pedro MATERNITE RED EARTH

Gbokle-Nawa-San PedroSan-Pedro AIBEF

Gbokle-Nawa-San Pedro Soubre Okrouyo Urban Health Centre

Gbokle-Nawa-San Pedro Soubre YABAYO Urban Health Centre

Gbokle-Nawa-San Pedro Soubre Meagui Urban Health Centre

Gbokle-Nawa-San Pedro Soubre Grand Zattry Urban Health Centre

Gbokle-Nawa-San Pedro Méagui Sante Oupoyo Health Centre

Gbokle-Nawa-San Pedro Taboo CSR MENEKE

Gbokle-Nawa-San PedroTabou Olodio Urban Health Centre

Gbokle-Nawa-San Pedro Buyo Buyo General Hospital

Gbokle-Nawa-San Pedro Buyo Urban Health Centre Buyo-Plateau

ABIDJAN2 Treichville-MarcoryService of Gyneco-obstetrics CHU Treichville

ABIDJAN2 Treichville-MarcoryFSU Com Anomabo

ABIDJAN2 Treichville-MarcoryCSU Com Arras3

ABIDJAN2 Treichville-MarcoryKO 'KHOUA National Blood Transfusion Centre

ABIDJAN2 Treichville-MarcoryTreichville Tuberculosis Centre

ABIDJAN2 Treichville-MarcoryTreichville General Hospital

ABIDJAN2 Treichville-MarcoryAnti venereal disease clinic INHP

ABIDJAN2 Treichville-MarcoryCentre Médical la Pierre Angulaire

ABIDJAN2 Treichville-MarcoryAIBEF

ABIDJAN2 Treichville-MarcorySMIT

ABIDJAN2 Treichville-MarcoryCIRBA

ABIDJAN2 Treichville-MarcoryPPH Treichville

**Advanced strategy (10 Districts)**

**Partner Region Districts**

APROSAM GNSP San Pedro

GNSP Taboo

Orasur GNSP Soubré

GNSP Méagui

BLETY Abidjan 1 Youpougon East

Eloé South Comoé Aboisso

South Comoé Aboisso

Espace confianceAbidjan 2 Port Bouët -Koumassi

Abidjan 2 Treichville-Marcory

South Comoé Adiaké

South Comoé Grand Bassam

Red Ribbon Abidjan 1 Youpougon East

**Mali :**

**Fixed strategy (33 sites)**

**Region Clinics/Centres**

Sikasso Sikasso Hospital

USAC CSRef Koutiala

Sikasso Sexual Health Unit

CCDV Yanfolila

Koulikoro USAC CSRef Kati

USAC CSRef Fana

CCDV Kati

CCDV Ouéléssébougou

CCDV Kangaba (Kourémalé)

Kayes Kayes Hospital

USAC CsRef Kayes

CCDV Kayes

CCDV Kéniéba

Bamako HPG

HGT

MCH

CESAC

CNAM

USAC CsRef Common 1 to 6

Halles de Bamako

CCDV Bamako

Ségou Ségou Hospital

Support site for the NGO Walé

Sikasso Sexual Health Unit

CCDV Niono

CCDV Bougouni

CCDV Kolondièba

CCDV Sélingué,

**Advanced Strategy (23 Districts)**

**Partner Region Districts**

ARCAD SIDA Sikasso Sikasso, Koutiala

Bamako Bamako

Segou Ségou

SOUTOURA Bamako Bamako

Segou Niono

Sikasso Bougouni, Kolondièba, Sélingué, Yanfolila

Kayes Kayes, Kéniéba

Koulikoro Koulikoro

DANAYASO Bamako Bamako

Segou Segou

Sikasso Sikasso, Koutiala, Koumentou

AKS Sikasso Sikasso, Koutiala

SAHELKayes AMPRODE Kayes, Nioro, Diéma, Kita, Bafoulabé, Kéniéba

Koulikoro Koulikoro, Kati, Kalaban Coro, Fana, Diola

**Senegal :**

**Fixed strategy (19 sites)**

**Region Districts - Clinics/Centres**

Dakar Guédiawaye, Las palmas

Dakar Guédiawaye, King Baudouin adult

Dakar Diamniadio, PS Sébikotane

Dakar Rufisque, CS Rufisque

Dakar Rufisque, EPS Rufisque

Dakar Pikine, CS Baye Talla Diop

Dakar Pikine, Pikine

Dakar South, polyclinic/ IHS

Dakar Mbao, CS Mbao

DAKAR Centre, CRCF

DAKAR Centre, CTA

THIES Thiès, CS of Thiès (10th)

THIES EPS1 Mbour, EPS1 Mbour

THIES Thiès, CHR Thiès

Ziguinchor Diouloulou, PS Kaffountine

Ziguinchor Diouloulou, CS Diouloulou

Ziguinchor Ziguinchor, CHR Ziguinchor

Ziguinchor Ziguinchor, CS Ziguinchor

Ziguinchor Bignona, CS Bignona

**Advanced Strategy (14 Districts)**

**Partner Region Districts**

ENDA Health DAKAR Centre, West, North, South,

Diamniadio, Pikine, Guédiawaye, Keur Massar

Mbao, Rufisque, Thiaroye

THIES Thiès and Mbour

Ziguinchor Ziguinchor

CEPIAD DAKAR Centre, West, North, South

Pikine, Guédiawaye, Keur Massar, Mbao, Thiaroye, Rufisque

THIES Mbour

**WP Key Populations**

Information leaflet

Key Population Survey

Individual interview

# ATLAS: HIV Self-Test, Free Access to Know Your HIV Status

Version 2.1 of 5 August 2019 approved by the WHO Research Ethics Committee (date) and the ethics committees of Côte d'Ivoire (opinion, date), Mali (opinion, date) and Senegal (opinion, date).

**Principal investigators**: Alice Desclaux and Odette Ky-Zerbo, TransVIHMI/Institut de Recherche pour le Développement (IRD), Montpellier, France. Contact: kyzerbo_odette@yahoo.fr

**Responsible for data processing**: Institut de Recherche pour le Développement, 44 bd de Dunkerque, Marseille, France. Contact: dpo@ird.fr

**Financeur** : Unitaid, Global Health Campus, Chemin du Pommier 40, 5th floor, 1218 Grand-Saconnex, Geneva, Switzerland. Tel. +41 22 791 12 00

## Research in the context of the ATLAS programme

Following the recommendations of the World Health Organization (WHO) and the experience gained in East and Southern Africa, Unitaid wanted to promote and provide HIV self-tests in West Africa through the funding of the ATLAS project in Côte d'Ivoire, Mali and Senegal, led by a consortium composed of the NGO Solthis (leader) and the Institute of Research for Development (IRD) for the research part.

The aim of this study is to adapt the provision of HIV self-testing to make it more accessible, acceptable and effective for key populations such as sex workers, men who have sex with men and drug users. To this end, we would like to hear your views on HIV self-testing.

## Procedures

The interview consists of an individual interview that will last approximately 45 min-1 hour. You have been identified to take part in this study because you belong to one of the key populations mentioned/you have used a self-testing/you are a key player in the field of HIV testing. If you agree to participate, you will be asked questions about your knowledge and opinions about self-testing. And if you have already done a self-test, you will be asked questions about how it was done and whether there were any problems with the test. Our conversation will be recorded with this audio device if you agree to it, so that we can be reminded of what you said later.

Your answers will remain confidential. No one outside the research team will have access to the personal information you give us. All files will be kept confidential. In the documents, we will use codes instead of your name or the name of any person or organisation you may mention. If, at the end of the study, other researchers wish to have access to the information you have given us for other HIV surveys, they will have to ask us first. If we agree in view of their project, they will have to sign a confidentiality agreement and they will see an anonymised transcript of this interview in which all passages that might recognise you have been removed.

## Your participation in this study is entirely voluntary

You are free to withdraw or not answer the questions at any time you wish. There will be no adverse consequences if you decide not to participate or if you decide to withdraw before the end of the discussion.

## What are the risks you face if you participate in this study?

In our opinion, there are no major risks associated with participating in this study. The only risk we see in this study is social, i.e. that someone outside the study may find out about your status or practices/behaviours through participation in this study. But we minimise this risk by interviewing you in a confidential setting. Therefore we will not mention your name anywhere and all audio recordings will be destroyed at the latest at the end of the study.

## What are the benefits of participating in this study?

There are no individual benefits to participating in this study, but your participation will enable us to better understand how to organise the provision of self-testing for sex workers, men who have sex with men and drug users, and thus to participate in the implementation of self-testing for HIV in your country. At the end of the interview, if you wish, we will refer you to the appropriate associations or medical referrals for information/care or appropriate treatment.

## Compensation

You will not be paid for your participation but if you agree to participate in this study, at the end of your participation you will receive a sum of XXX (to be completed according to the ethical practices of the country) to compensate for your travel expenses.

## Data processing

The data collected is the data you will give us during the interview. You are free not to answer one or more questions that you do not wish to answer. Your data will be analysed in relation to the objectives of this research. You have a right of access, a right to rectify your collected data, the right to object (right to object) and the right to limit their use (right to limit processing). These rights can be exercised by contacting the interviewer or the principal investigator of the study by giving the interview number. Finally, your personal data will be kept for the time necessary for their analysis and the publication of the results (maximum 5 years after the end of the project) and then your anonymised data will be archived in accordance with the regulations in force for a long period of time.

## If you have any questions

If you have any doubts or if you need further clarification, you can contact the mediator of this study at this number: tel. no. __XXXXXXXXXXXX

## Your rights as a participant

Again, your participation is voluntary and I repeat that you are free to change your mind at any time regarding your participation or to refuse to answer certain questions. This study has been reviewed and approved by the Ethics Committees of Cote d'Ivoire, Mali and Senegal, and the WHO Ethics Committee.

You have the right to keep a copy of this consent form. If you have any questions about how you are treated in the study or your rights as a participant, you can contact the Ethics Committee that has agreed to the study in your country at the following address __________________________________________________________________Tel: XXXXXXXXXXXXXXXXXXXXXXXXXXXXXXXXXX

At the end of this study, you will be able to get the results from XXX (local NGO). Also a website dedicated to the ATLAS programme has been opened [(](https://atlas.solthis.org/)https://atlas.solthis.org/). You can find this information note as well as the results of the research at the end of the project.

**Date on which the information was issued:** I__I__I / I__I__I / I__I__I

**By:** (surname, first name, position)

Consent Form

Key Population Survey

Individual interview

Version 2.1 of August 5, 2019

**Principal investigators**: Alice Desclaux and Odette Ky-Zerbo, TransVIHMI/Institut de Recherche pour le Développement (IRD), Montpellier, France. Contact: kyzerbo_odette@yahoo.fr

Identification number --------------------------------------------

The information leaflet, v2.0 of 22 July 2019, describing the purpose, methods, benefits and risks of qualitative research (individual interviews) on HIV self-testing was read and explained to me. I had the opportunity to have satisfactory answers to all the questions about the study and I had enough time to think about my participation.

□ I voluntarily agree to participate.

□ I agree to be registered. □ I do not agree to be registered.

_____________________________________________________________________

Participant's signature or fingerprint Date

I certify that the nature and purpose, potential benefits and possible risks of participating in this research have been explained to the participant above.

________________________________________  ____________________

Signature of the Person having collected the Consent Date

Information leaflet

Key Population Survey

Individual telephone interview

# ATLAS: HIV Self-Test, Free Access to Know Your HIV Status

Version 2.1 of 5 August 2019 approved by the WHO Research Ethics Committee (date) and the ethics committees of Côte d'Ivoire (opinion, date), Mali (opinion, date) and Senegal (opinion, date).

**Principal investigators**: Alice Desclaux and Odette Ky-Zerbo, TransVIHMI/Institut de Recherche pour le Développement (IRD), Montpellier, France. Contact: kyzerbo_odette@yahoo.fr

**Responsible for data processing**: Institut de Recherche pour le Développement, 44 bd de Dunkerque, Marseille, France. Contact: dpo@ird.fr

**Financeur** : Unitaid, Global Health Campus, Chemin du Pommier 40, 5th floor, 1218 Grand-Saconnex, Geneva, Switzerland. Tel. +41 22 791 12 00

## Research in the context of the ATLAS programme

Following the recommendations of the World Health Organization (WHO) and the experience gained in East and Southern Africa, Unitaid wanted to promote and deploy HIV self-tests in West Africa through the funding of the ATLAS project in Côte d'Ivoire, Mali and Senegal, led by a consortium composed of the NGO Solthis (lead partner) and the Institut de Recherche pour le Développement (IRD) for the research part.

The aim of this study is to adapt the offer of HIV self-testing to make it more accessible, acceptable and effective for sex workers, men who have sex with men and drug users. To this end, we would like to hear your views on HIV self-testing.

## Procedures

This research consists of an individual interview that will last approximately 45mn-1h. You have been selected because you called the toll-free number to answer the "voucher" survey and used an HIV self-test.

If you agree to participate, you will be asked questions about your knowledge and opinions about self-testing. And you will be asked questions about how it went and whether there were any problems with the test. Our conversation will be recorded if you agree, so that we can be reminded of what you said later.

Your answers will remain confidential. No one outside the research team will have access to the personal information you give us. All files will be kept confidential. In the documents, we will use codes instead of your name or the name of any person or organisation you may mention. If, at the end of the study, other researchers wish to have access to the information you have given us for other HIV surveys, they will have to ask us first. If we agree in view of their project, they will have to sign a confidentiality agreement and they will see an anonymised transcript of this interview in which all passages that might recognise you have been removed.

## Your participation in this study is entirely voluntary

You are free to withdraw or not answer the questions at any time you wish. There will be no consequences if you decide not to participate or if you decide to withdraw before the end of the interview.

## What are the risks you face if you participate in this study?

In our opinion, there are no major risks associated with participating in this study. The only risk we see in this study is social, i.e. that someone outside the study may find out about your status or practices/behaviours through participation in this study. But we minimise this risk by interviewing you in a confidential setting. Therefore we will not mention your name anywhere and all audio recordings will be destroyed at the latest at the end of the study.

## What are the benefits of participating in this study?

There are no individual benefits to participating in this study, but your participation will give us a better understanding of how to organise the provision of self-testing for sex workers, men who have sex with men and drug users, and thus participate in setting up the provision of HIV self-testing in your country.

## Compensation

You will not be paid for your participation in the study.

## Data processing

The data collected is the data you will give us during the interview. You are free not to answer one or more questions. Your data will be analysed in relation to the objectives of this research. You have a right of access, a right to rectify your collected data, the right to object (right of opposition) and the right to limit their use (right to limit processing). These rights can be exercised by contacting the investigator or the principal investigator by giving the number of the interview. Finally, your personal data will be kept for the time necessary for their analysis and the publication of the results (maximum 5 years after the end of the project) and then your anonymised data will be archived in accordance with the regulations in force for a long period of time.

## If you have any questions

If you have any doubts or if you need further clarification, you can contact the mediator of this study at this number: tel. no. __XXXXXXXXXXXX

## Your rights as a participant

Again, your participation is voluntary and I repeat that you are free to change your mind at any time regarding your participation or to refuse to answer certain questions. This study has been reviewed and approved by the Ethics Committees of Cote d'Ivoire, Mali and Senegal, and the WHO Ethics Committee.

You have the right to keep a copy of this consent form. If you have any questions about how you are treated in the study or your rights as a participant, you can contact the Ethics Committee that has agreed to the study in your country at the following address __________________________________________________________________Tel: XXXXXXXXXXXXXXXXXXXXXXXXXXXXXXXXXX

At the end of this study, you will be able to find out the results from XXX (local NGO?). Also a website dedicated to the ATLAS programme has been opened [(](https://atlas.solthis.org/)https://atlas.solthis.org/). You will be able to find this information notice as well as the results of the research at the end of the project.

**Date on which the information was issued:** I__I__I / I__I__I / I__I__I

**By:** (surname, first name, position)

Consent Form

Key Population Survey

Individual telephone interview

Version 2.1 of August 5, 2019

**Principal investigators**: Alice Desclaux and Odette Ky-Zerbo, TransVIHMI/Institut de Recherche pour le Développement (IRD), Montpellier, France. Contact: kyzerbo_odette@yahoo.fr

*To be completed by the interviewer*

Identification number --------------------------------------------

I certify that the nature and purpose, potential benefits and possible risks of participating in this research have been explained to the participant.

The information leaflet, v2.0 of 22 July 2019, describing the purpose, methods, benefits and risks of qualitative research (individual interviews) on HIV self-testing was read and explained. The participant had the opportunity to have satisfactory answers to all questions about the study and had enough time to think about my participation.

□ The participant voluntarily agrees to participate.

□ The participant agrees to be registered.

□ The participant does not agree to be registered.

I certify that the nature and purpose, potential benefits and possible risks of participating in this research have been explained to the participant.

________________________________________  ____________________

Signature of the Person having collected the Consent Date

Information leaflet

Key Population Survey

Group discussions

# ATLAS: HIV Self-Test, Free Access to Know Your HIV Status

Version 2.1 of 5 August 2019 approved by the WHO Research Ethics Committee (date) and the ethics committees of Côte d'Ivoire (opinion, date), Mali (opinion, date) and Senegal (opinion, date).

**Principal investigators**: Alice Desclaux and Odette Ky-Zerbo, TransVIHMI/Institut de Recherche pour le Développement (IRD), Montpellier, France. Contact: kyzerbo_odette@yahoo.fr

**Responsible for data processing**: Institut de Recherche pour le Développement, 44 bd de Dunkerque, Marseille, France. Contact: dpo@ird.fr

**Financeur** : Unitaid, Global Health Campus, Chemin du Pommier 40, 5th floor, 1218 Grand-Saconnex, Geneva, Switzerland. Tel. +41 22 791 12 00

## Research in the context of the ATLAS programme

Following the recommendations of the World Health Organization and the experience gained in East and Southern Africa, Unitaid wanted to promote and deploy HIV self-tests in West Africa through the funding of the ATLAS project in Côte d'Ivoire, Mali and Senegal, led by a consortium composed of the NGO Solthis (lead partner) and the Institut de Recherche pour le Développement (IRD) for the research part.

The aim of this study is to adapt the provision of HIV self-testing to make it more accessible, acceptable and effective for key populations such as sex workers, men who have sex with men and drug users. To this end, we would like to hear your views on HIV self-testing.

## Procedures

You have been identified to take part in this study because you either belong to one of the groups mentioned in the previous paragraph and/or you have used an HIV self-test.

This consists of a group discussion that will last about 1h30mn. If you agree to participate, you will be asked questions about your knowledge and opinions about self-testing. And if you have already taken a self-test, you will be asked questions to find out how it went and if there were any problems with the test. We will not try to find out your HIV test result during the group discussion. There will be 8-10 participants in this discussion. Our conversation will be recorded with an audio device so that we can remember what you told us later.

To ensure confidentiality, you will be assigned a number for the discussion and you will begin by saying your number when you speak. Your answers will remain confidential. The paper on which you will sign (indicating your consent) will be kept separate from your answer. No one outside the research team will have access to the personal information you give us. All files will be kept confidential. In the documents, we will use codes instead of your name or the name of any person or organisation you may mention. If, at the end of the study, other researchers wish to have access to the information you have given us for other HIV surveys, they will have to ask us first. If we agree in view of their project, they will have to sign a confidentiality agreement and they will see an anonymised transcript of this interview in which all passages that might recognise you have been removed.

## Your participation in this study is entirely voluntary

You are free to withdraw or not answer the questions at any time you wish. There will be no consequences if you decide not to participate or if you decide to withdraw before the end of the discussion.

## What are the risks you face if you participate in this study?

In our opinion, there are no major risks associated with participating in this study. The only risk we see in this study is social, i.e. that someone outside the study may discover your practices/behaviours through participation in this study. But we minimise this risk by interviewing you in a confidential setting. Also we will not mention your name anywhere and all audio recordings will be destroyed at the end of the study. And because you are in a group, someone in the group might be tempted to report back to the community about your practices/behaviours or what you said during the discussion. We will ask all participants to agree not to disclose after the discussion what you have heard or seen during the group discussion.

## What are the benefits of participating in this study?

There are no individual benefits to participating in this study, but your participation will enable us to better understand how to organise the offer of self-testing to sex workers, men who have sex with men, drug users and thus participate in the implementation of the offer of HIV self-testing in your country. At the end of the group discussion, if you wish, we can refer you to the appropriate associations or medical referents for information/care or appropriate treatment.

## Compensation

You will not be paid for this study, but if you agree to participate in this study, at the end of your participation you will receive a sum of _(to be completed according to the ethical practices of the country) to compensate for your travel expenses.

## Data processing

The data collected is the data you will give us during the interview. You are free not to answer one or more questions that you do not wish to answer. Your data will be analysed in relation to the objectives of this research. You have a right of access, a right to rectify your collected data, the right to object (right to object) and the right to limit their use (right to limit processing). These rights can be exercised by contacting the interviewer or the principal investigator by giving the interview number and your number in the discussion. Finally, your personal data will be kept for the time necessary for their analysis and the publication of the results (maximum 5 years after the end of the project) and then your anonymised data will be archived in accordance with the regulations in force for a long period of time.

## If you have any questions

If you have any doubts or if you need further clarification, you can contact the mediator of this study at this number: tel. no. __XXXXXXXXXXXX

## Your rights as a participant

Again, your participation is voluntary and I repeat that you are free to change your mind at any time regarding your participation or to refuse to answer certain questions. This study has been reviewed and approved by the Ethics Committees of Côte d'Ivoire, Mali and Senegal, and the WHO Ethics Committee.

You have the right to keep a copy of this consent form. If you have any questions about the way you are treated in the study or your rights as a participant, you can contact the Ethics Committee that has agreed to the study in your country at the following address: T__________________________________________________________________XXXXXXXXXXXXXXXXXXXXXXXXXXXXXX

At the end of this study, you will be able to get the results from XXX (local NGO). Also a website dedicated to the ATLAS programme has been opened [(](https://atlas.solthis.org/)https://atlas.solthis.org/). You will be able to find this information notice as well as the results of the research at the end of the project.

**Date on which the information was issued:** I__I__I / I__I__I / I__I__I

**By:** (surname, first name, position)

Consent Form

Key Population Survey

Group discussion

Version 2.1 of August 5, 2019

Identification number ----------------------------------

**Principal investigators**: Alice Desclaux and Odette Ky-Zerbo, TransVIHMI/Institut de Recherche pour le Développement (IRD), Montpellier, France. Contact: kyzerbo_odette@yahoo.fr

The information leaflet, Version 2.1 of 5 August 2019, describing the purpose, methods, benefits and risks of qualitative research (focus groups) on HIV self-testing was read and explained to me. I had the opportunity to have satisfactory answers to all the questions about the study and had enough time to think about my participation in the study.

□ I voluntarily agree to participate.

□ I agree to be registered. □ I do not agree to be registered (*if you refuse registration, you will not be able to take part in the study*).

□ I undertake not to divulge what I have heard/seen during the discussion.

_____________________________________________________________________

Participant's signature or fingerprint Date

I certify that the nature and purpose, potential benefits and possible risks of participating in this research have been explained to the participant above.

________________________________________  ____________________

Signature of the Person having obtained Consent Date

WP Key Populations

INTERVIEW GUIDES

QUALITATIVE STUDY

Version 2.1 of August 5, 2019

## Survey 1. ADVIH device and factors (Key Actors)

## In-depth individual interview guide

**Respondent identification number ______________________________**

**Description of the respondent**

Country

Locality

Respondent category

Marital status

Level of schooling

Profession

Structure

Approximate age

Sex

**Interviews**

| **Themes** | **Questions** | **Comments** |
| --- | --- | --- |
| General | ∎ Tell us about the situation and evolution of the HIV epidemic in Côte d'Ivoire, Mali, Senegal (choose the country indicated)  ∎ Is the epidemic the same in all parts of the country? Are some regions more infected than others?  ∎ From your point of view, which groups are more affected (men/women, key populations/general population, children/youth/adults)? |  |
| Reaching the first 90 | ∎Comment do you appreciate the level of achievement of the first 90 in (COUNTRY: choose the country indicated]?  ∎Pour For you, what justifies this level of screening in your country?  ∎ Which populations have the least access to HIV testing? How do you explain this? |  |
| Perceptions of **ADVIH** as a screening strategy | ∎Parlez do we have screening strategies implemented in [COUNTRY] for the general population; key populations? Limitations and benefits of each strategy?  ∎Que do you think about **ADVIH**? Have you tried it yourself? In general, does it remind you of other self-tests?  ∎Que do you think about the introduction of **ADVIH** as a screening strategy in the country (for/against, reasons...)? Among key populations?  ∎Avantage comparison of **ADVIH in** relation to other strategies |  |
| **ADVIH in the framework of the ATLAS project**: opportunities and obstacles to the introduction of **ADVIH and** its support system | ∎ From your point of view, what could lead key populations to accept **ADVIH (**try to find out whether the motivations are the same from one key population to another)?  ∎Qu 'what might prevent them from doing so (i.e. whether barriers are the same across key populations)?  ∎Qu could facilitate the implementation of this project in (COUNTRY) at the political? social? health level?  ∎Quelles are the difficulties that the project is likely to face at the political? social? health? level. | Take stock of the respondent's knowledge of the ATLAS project activities. And provide him/her with additional information before asking the various questions. |
| **ADVIH in the framework of the ATLAS project**: opportunities and obstacles in the country's associative and health system | ∎Quels could be the benefits of ADVIH for the health system? for NGOs and associations involved in the fight against HIV?  ∎Qu Could this be what is preventing a good supply of ADVIH in health facilities? By NGOs and associations?  ∎ To health workers and community actors involved in the delivery of ADVIH  ∎Dites how does the introduction of ADVIH make it easier for you to offer screening to key populations?  ∎ How does this make it difficult for you to offer screening to key populations? What could help you? |  |
| ADVIH in the ATLAS project: opportunities and obstacles specific to each population | ∎ In your opinion, to whom (which population) should ADVIH be offered as a priority? Why  ∎quelle is the population for whom the provision of ADVIH is easiest? What explains this?  ∎ What is the population in which dispensing is difficult? What explains this?  ∎ In your opinion, are there risks of abuse, and if so, what are they?  ∎Craignez do you have people being forced to take the test against their will? Under what circumstances?  ∎ If coercive practices exist, how can victims report them and protect themselves from them?  ∎Population key by key population: what are the difficulties that actors may encounter in offering HIV and AIDS to this population? Difficulties that the key population concerned may encounter in dispensing to peer? partners? What are the difficulties that the key population may encounter in the self-administration of the supervised test? and in the self-administration of the unsupervised test? |  |
| Perceptions of the support system (advice, green line, tools) | ∎ The project uses secondary providers of kits for key populations; who do you think can play this role in TS, MSM and UD settings (gender, age, profession)?  ∎ How do you appreciate the system in place to give you advice to people who carry out ADVIH: face-to-face advice, green line (relevance, ease of access, quality of advice/responses, effectiveness, sustainability)?  ∎ What do you suggest instead or in addition? |  |
| Link with care services | ∎ What do you think of the current referral system based on telephone counselling?  ∎ In your opinion, what can we do to help self-tested people who have a positive result to use the facilities for confirmation? care? |  |
| Recommended adjustments for PCs. | ∎ In your opinion, what information should be given to key populations prior to self-testing? And after the result (explore for both negative and positive results)?  ∎ Or could HIV-AIDS be most accessible for TS, MSM and UD? At what point should ADVIH be offered to these different populations? |  |

## Survey 2. Motivations and Obstacles (PS, MSM, CDI)

## Group discussion guide

**Respondent identification number**

1. _______________
2. _______________
3. _______________
4. _______________
5. _______________
6. _______________
7. _______________
8. _______________

**Description of respondents**

Country

Locality

Population concerned _________________________________

| **Number** | **Sex** | **Age** | **Profession** | **Marital status** | **Last class attended** |
| --- | --- | --- | --- | --- | --- |
|  |  |  |  |  |  |
|  |  |  |  |  |  |
|  |  |  |  |  |  |
|  |  |  |  |  |  |
|  |  |  |  |  |  |
|  |  |  |  |  |  |
|  |  |  |  |  |  |

**Social background of respondents** (inter-knowledge of respondents, same workplace, etc.)

**Interviews**

| **Themes** | **Questions** | **Comments** |
| --- | --- | --- |
| Knowledge and perceptions of ADVIH | ∎Avant to talk about ADVIH, tell us about your experience of using commonly available testing services (*perceptions of strengths and weaknesses to be collected*)  ∎Que do you know about ADVIH?  ∎ Compared to routine screening services, what are the advantages of ADVIH for you?  ∎ What are the specific advantages of [PC CATEGORY]?  ∎ What are the disadvantages of ADVIH?  ∎ Are there any disadvantages that are specific to your community (violence, coercion...)?  ∎Selon What information should be given to an individual prior to self-administration of the test?  ∎ And after the result (*explore for both negative and positive results*)?  ∎ How should he be given this information (sufficient notice? a number he can call? a physical presence?) Who should give this information?  ∎ Have you used ADVIH for yourself?  ∎ Did you offer it to a third party? |  |
| Motivations and obstacles for ADVIH | ∎ In your opinion, what are the reasons that can lead TS, MSM, UD to make a PLWHA? To prefer it to a routine test?  ∎ Why would they refuse it?  ∎ Would you agree to do an ADVIH if you were offered one? Would you prefer to do it alone or in the presence of a professional? Why/why not?  ∎ Would you be willing to propose it to a friend? Why/why not? On what condition would you propose it? Why not? Do you think there are any steps that should be taken before offering ADVIH to him/her?  ∎ Would you be willing to offer it to a sexual partner? Why/why not? On what condition would you offer it? Why not? Do you think that there are steps to be taken before offering him/her ADVIH?  ∎ *To TS only* : What type of partner would you be more comfortable proposing it to: regular client, occasional client, spouse? Why is that? Under what conditions would you propose it? Why not? Do you think that there are steps to be taken before offering him/her ADVIH? |  |
| Confirmation of test result | ∎ *If an ADVIH is positive, you have to go to a facility to confirm that you are really infected with HIV*: do you think your peers would go to a facility to confirm that they are infected with HIV? Why/why not? What would help them to do this? What difficulties would they have to face in order to make this confirmation? |  |
| ADVIH offer device | ∎ Apart from health personnel, who do you think could be the provider of screening kits in TS, MSM, UD settings?  ∎ Where might HIV and AIDS be most accessible for TS, MSM and UD? When should PLWHAs be made available to these different populations? |  |
| Suggestions for promoting the practice of ADVIH for each key population in each country | From your point of view, what should be done to improve membership of ADVIH :  -from MSM to [COUNTRY]?  -from TS to [COUNTRY]?  -from SUs to [COUNTRY]. |  |

## Survey 3. Experience of ADVIH users

## In-depth individual interview guide

**Respondent identification number ______________________**

**Ask for oral consent if by telephone - written consent if face-to-face**

**Description of the respondent**

Country

Locality

Respondent category

Marital status

Level of schooling

Profession

Structure

Approximate age

Sex

**Interviews**

| **Theme** | **Questions** | **Comments** |
| --- | --- | --- |
| Recourse to ADVIH (motivations, circumstances, screening routes before ADVIH)  These are the facts | ∎ Can you give me the reasons why you did your last test?  ∎ Why did you choose to do it through an ADVIH?  ∎ Tell me how it went at the test site: How did it feel to do the test itself?  ∎ ADVIH supervised or not? what relationship with the person who gave it to him/her? staff attitudes, advice?  ∎ Did you need to speak with someone before the test (green line or peer)? If yes, the reasons and the questions they wanted to elucidate before the test to explore  ∎ And after the test, did you need to talk to someone (green line or peer)? If so, what were the reasons and the questions they wanted to elucidate before the test to explore? |  |
| Opinions on the test (perceptions of the process, satisfaction)    Here are some opinions | ∎ What do you think of the testing kit (clarity of instructions, clarity of results, how the kit is packaged)  ∎ What information should you have been given before the self-test?  ∎ How would you have liked to have this information (sufficient notice? a number to call? a physical presence?) Who should give this information?  ∎ What information should have been given to you after the test)?  ∎ How would you have liked to have this information (sufficient notice? a number to call? a physical presence?) Who should give this information? |  |
| Social experience (social context of implementation, relationship with the proposer, violence or coercion suffered/exercised on the partner/girlfriend, stigmatisation, abuse, changes in terms of prevention strategies and social relationships) | ∎ How did you find out about ADVIH: who told him/her about it, under what circumstances, why he/she told him/her about it? Exactly what did he/she tell him/her?  ∎ Tell me how it happened, from making the decision to knowing the HIV outcome: did he/she decide himself/herself whether to do the ADVIH or not? If the decision was imposed on him/her, who imposed it on him/her and why? Did he/she do it alone, or was it a professional or someone other than the professionals who tested him/her?  ∎ How did you react to the result?  ∎ What has knowing your status changed in your life (changes in terms of prevention strategies and social relations)?  ∎ Do you ever offer ADVIH to peers? In general, how do they react when you offer them ADVIH? Have you had any cases of violence against you? What were the person's motivations and how did you manage the situation?  ∎ Do you ever offer ADVIH to sexual partners? In general, how do they react when you offer them ADVIH? Have you had any cases of violence against you? What were the person's motivations and how did you manage the situation? |  |
| Care itineraries (persons of recourse, test confirmation itineraries, time taken for confirmation, access to care) | ∎ How did you react when you saw a positive result? With whom did you talk about it? What did you tell yourself?  ∎ Where did you go for test confirmation? How long after the first result, did you carry out the confirmation? How did it go at the confirmation site (staff attitudes, counselling, testing, care/referral for care) |  |
| Experience of the ADVIH result (Psychological reactions, sharing of results, reactions of the entourage, behavioural changes induced, other social effects) | ∎ How was your result announced to you?  ∎ How did you feel when you discovered your result? Did you tell anyone about it? Who was it? How did this person react?  ∎ What has knowing your HIV status through this test changed in your life (relationships with spouse, partner, clients for TS; other sexual behaviours and practices)? |  |
| Perception of the system (satisfaction with the process, support and information received, needs expressed, suggestions). | Overall, are you satisfied with the way things went (process, support and information received)? What worked well? What needs to be improved? Is the ADVIH, as provided, suitable for TS, MSM, UD? |  |
| Difficulties and satisfaction (access to ADVIH, achievement, suggestions) | ∎ In your opinion, how easy/difficult is it to take the test yourself (did you feel comfortable taking the test yourself? What was difficult? What was easy?)  ∎ What difference do you make between ADVIH and HIV testing in routine services?  ∎ Compared to routine screening services, what do you think of ADVIH?  ∎ On the whole, are you satisfied with having done ADVIH? What are you satisfied with? What did you find the hardest (access? self-administering the test?)? |  |
| Suggestions/Recommendations | What suggestion would you make for :  -improve the ATVIH screening kit,  -Bring more TS, MSM, UD to join ADVIH,  -encourage those who have a positive result to use the structures for confirmation, to access care? |  |

### Survey 4. Assessment and proposals (Key actors)

### In-depth individual interview guide

**Respondent identification _____________________________**

**Description of the respondent**

Country

Locality

Respondent category

Marital status

Level of schooling

Profession

Structure

Approximate age

Sex

**Interviews**

| **Themes** | **Questions** | **Comments** |
| --- | --- | --- |
| Perceptions of ADVIH as a screening strategy | ∎ For more than two years, ADVIH has been used as a screening strategy in your country. What has this changed? | Change for key populations, PS clients, health services, community-based organisations, national screening policy |
| **ADVIH in the framework of the ATLAS project**: opportunities and obstacles to the introduction of ADVIH and its support system | ∎ From your point of view, what were the motivations of key populations to accept ADVIH (key population by population)?  ∎ What obstacles have key populations faced? |  |
| **ADVIH in the framework of the ATLAS project**: opportunities and obstacles in the country's associative and health system | ∎ What were the benefits for the health system? for NGOs and associations involved in the fight against HIV?  ∎ What has been a major obstacle to good provision of ADVIH in health facilities? By NGOs and associations?  Health workers and community actors involved in the provision of HIV-related AIDS services.  ∎ Tell us how the introduction of ADVIH has made it easier for you to offer screening to key populations?  ∎ How was this difficult for you? |  |
| ATHIV in the ATLAS project: opportunities and obstacles specific to each population | ∎ Who is the population to whom the provision of HIV-AIDS has been easiest? What explains this?  ∎ What is the population in which you have had the most difficulties? What explains this?  *Key population by key population*: what difficulties have the actors encountered? Difficulties encountered by the key population concerned in peer? partner dispensing? What are the difficulties encountered in the supervised self-administration of the test? and in the unsupervised self-administration of the test? |  |
| Perceptions of the support system (advice, green line, tools) | How do you appreciate the system set up to give you advice to the people who carry out ADVIH: face-to-face advice, green line (relevance, ease of access, quality of advice/responses, effectiveness, sustainability)? What do you suggest instead? |  |
| Integration of the system and impact on the healthcare system | ∎ From your point of view, can ADVIH be considered as a screening strategy like routine screening in [COUNTRY]?  ∎ How has the implementation of the ADVIH project had an impact on the health system? On screening [COUNTRY]?  ∎ What was the contribution of the Ministry of Health in the implementation of the ADVIH project? In your opinion, is there anything else that the Ministry of Health could have done as part of the ADVIH project? |  |
| Challenges in the provision of ADVIH to PCs |  |  |

**WP Screening of index cases**

WP Screening of index cases

Information leaflet

# ATLAS: HIV Self-Test, Free Access to Know Your HIV Status

Version 2.1 of 5 August 2019 approved by the WHO Research Ethics Committee (date) and the ethics committees of Côte d'Ivoire (opinion, date), Mali (opinion, date) and Senegal (opinion, date).

**Contact**: XXX

**Principal Investigators**: Dolorès Pourette and Sokhna Boye, CEPED/IRD, Paris, France. Contact: dolores.pourette@ird.fr

**Responsible for data processing**: Institut de Recherche pour le Développement, 44 bd de Dunkerque, Marseille, France. Contact: dpo@ird.fr

**Financeur** : Unitaid, Global Health Campus, Chemin du Pommier 40, 5th floor, 1218 Grand-Saconnex, Geneva, Switzerland. Tel. +41 22 791 12 00

## Research in the context of the ATLAS programme

Following the recommendations of the World Health Organization and the experience gained in East and Southern Africa, Unitaid wanted to promote and deploy HIV self-tests in West Africa through the funding of the ATLAS project in Côte d'Ivoire, Mali and Senegal, led by a consortium composed of the NGO Solthis (lead partner) and the Institut de Recherche pour le Développement (IRD) for the research part.

One of the objectives of the ATLAS programme is to evaluate the use of HIV self-tests to screen partners of people with HIV. This anthropological part of the programme aims to study the different issues involved in proposing to partners and the use of HIV self-tests by partners of people living with HIV.

## Procedures

You have been selected because you or your partner is a person living with HIV, or you are a health professional.

This research consists of an individual interview that will last approximately 1 hour. If you agree to participate, you will be asked questions about your knowledge and opinions about self-testing. And if you have already taken a self-test or offered it to your partner(s), you will be asked questions about how it went and whether there were any problems with the test. Our conversation will be recorded if you accept it with an audio device so that we can be reminded of what you said later.

Your answers will remain confidential. No one outside the research team will have access to the personal information you give us. All files will be kept confidential. In the documents, we will use codes instead of your name or the name of any person or organisation you may mention. If, at the end of the study, other researchers wish to have access to the information you have given us for other HIV surveys, they will have to ask us first. If we agree in view of their project, they will have to sign a confidentiality agreement and they will see an anonymised transcript of this interview in which all passages that might recognise you have been removed.

## Your participation in this study is entirely voluntary

You are free to withdraw or not answer the questions at any time you wish. There will be no consequences if you decide not to participate or if you decide to withdraw before the end of the discussion.

## What are the risks you face if you participate in this study?

In our opinion, there are no major risks associated with participating in this study. The only risk we see in this study is social, i.e. someone outside the study may find out about your status or practices/behaviours through participation in this study. But we minimise this risk by interviewing you in a confidential location, coding your data and destroying the records at the end of the project.

## What are the benefits of participating in this study?

## There are no individual benefits to participating in this study, but your participation will give us a better understanding of how to organise the offer of self-testing to partners of PvH and thus participate in the implementation of the offer of HIV self-testing in your country.

## Compensation

## You will not be paid for this study, but if you agree to participate in the study, at the end of your participation you will receive a sum of XXX (to be completed according to the ethical practices of the country) to compensate for your travel expenses.

## Data processing

The data collected is the data you will give us during the interview. You are free not to answer one or more questions. Your data will be analysed in relation to the objectives of this research. You have a right of access, a right to rectify your collected data, the right to object (right of opposition) and the right to limit their use (right to limit processing). These rights can be exercised by contacting the interviewer or the principal investigator of the study by giving your interview number. Finally, your personal data will be kept for the time necessary for their analysis and the publication of the results (maximum 5 years after the end of the project) and then your anonymised data will be archived in accordance with the regulations in force for a long period of time.

## If you have any questions

If you have any doubts or if you need further clarification, you can contact the mediator of this study at this number: tel. no. __XXXXXXXXXXXX

## Your rights as a participant

Again, your participation is voluntary and I repeat that you are free to change your mind at any time regarding your participation or to refuse to answer certain questions. This study has been reviewed and approved by the Ethics Committees of Cote d'Ivoire, Mali and Senegal, and the WHO Ethics Committee.

A website dedicated to the ATLAS programme has been opened [(](https://www.google.com/url?q=https://atlas.solthis.org/&sa=D&ust=1552897359532000&usg=AFQjCNFeeDHuOImRkHc0ycsy0zeHGUG_fQ)https://atlas.solthis.org/). You will be able to find this information notice as well as the research results at the end of the project.

You have the right to keep a copy of this consent form. If you have any questions about the way you are treated in the study or your rights as a participant, you can contact the Ethics Committee that has agreed to the study in your country at the following address__________________________________________________________________XXXXXXXXXXXXXXXXXXXXXXXXXXXXXX

At the end of this study, you will be able to find out the results from XXX (local NGO?).

**Date on which the information was issued:** I__I__I / I__I__I / I__I__I

**By:** (surname, first name, position)

WP Screening of index cases

Consent Form

Version 2.1 of August 5, 2019

**Principal Investigators**: Dolorès Pourette and Sokhna Boye, CEPED/IRD, Paris, France. Contact: dolores.pourette@ird.fr

The information leaflet, Version 2.1 of 5 August 2019, describing the purpose, methods, benefits and risks of qualitative research (individual interviews) on HIV self-testing was read and explained to me. I had the opportunity to have satisfactory answers to all the questions about the study and had enough time to think about my participation.

□ I voluntarily agree to participate.

□ I agree to be registered. □ I do not agree to be registered.

_____________________________________________________________________

Participant's signature or fingerprint Date

I certify that the nature and purpose, potential benefits and possible risks of participating in this research have been explained to the participant above.

________________________________________  ____________________

Signature of the Person having collected the Consent Date

OBSERVATION GUIDES

WP SCREENING FOR INDEX CASES

Version 2.1 of August 5, 2019

## Observation Guide - HIV Consultation

### General information

Location

Moment

Duration

Persons present

### ADVIH's proposal for the partner

Circumstances: "routine" or specific consultation

Information provided, terms used

### Responses of PLWHIV

Questions asked

Acceptance, refusal, request for time to think, more information?

Reasons expressed

### When the ADVIH kit is delivered

Description of the kit

Description of the information provided

Description of the questions asked / reactions of the patient

### Social report

Terms and conditions of exchanges

Attitudes and gestures

## 2. Observation Guide - Meetings Sites

##

### General information

Location

Type of activity

Moment

Duration

Persons present

### Specific information about the activity

Objective of the activity/order of the day

Profile of the participants

### Questions about HIV and AIDS

Are issues around HIV and AIDS addressed during staff meetings?

By whom?

How are they approached?

Participants' reactions

Positive/negative points discussed

Difficulties around the dispensary?

Negotiations on the terms and conditions of dispensation, the people to whom the self-test should be offered, the information to be provided, etc.

Discussions on HIV partner testing and how to provide self-tests to partners.

Discussions on confidentiality issues

WP SCREENING FOR INDEX CASES

THEMATIC MAINTENANCE GUIDES

Version 2.1 of August 5, 2019

## 1. Thematic interview guide - PvVIH

#### General information

Age

Activity

Place of residence

Family environment, marital status, number of children

Partner's activity

Education

Religion

Ethnicity

#### History of HIV

Duration since screening

Circumstances of screening

Medical follow-up and report to the care

Informed persons

Sharing information with the partner(s)

Transmission prevention practices (sexuality, PMTCT)

#### Knowledge about ADVIH

Sources of knowledge on ADVIH

Knowledge content

#### ADVIH's proposal to the partner

Have her describe how she was offered to deliver an ADVIH to her partner. Content of the information

Acceptance/refusal to offer the test to the partner

Expected reasons, motivations, benefits or limitations

If accepted :

Which partner (in case of multi-partnership)?

How was the proposal made to the partner (time, place, information content, media...)?

Reactions of the partner: acceptance to do the self-test, refusal, necessary time for reflection, seeking additional information, from whom (including the internet)?

Self-test practice: together / partner alone -, place, time...

Test result: sharing information with the person? With other people? At what time?

#### Impacts of ADVIH

On the relationship: trust, communication, distance, violence, sexuality...

On access and relationship to care: for the partner, for the person

On the relationship to HIV, to risk...

## 2. Thematic interview guide - PvVIH Partner

#### General information

Age

Activity

Place of residence

Family environment, marital status, number of children

Partner's activity

Education

Religion

Ethnicity

#### Knowledge about HIV

Sources of knowledge

Events

Modes of transmission

Prevention/Treatment/Cure

#### HIV prevention and testing (before ADVIH)

Attitudes towards HIV risk

Screening practices

Information on the serological status of relatives/partners

HIV communication

#### Knowledge and perceptions on ADVIH

Sources of knowledge on ADVIH

Knowledge content

Reactions during the proposal by the partner: have the partner describe how the proposal was made (terms used, place, time...), whether he or she felt sufficiently informed, whether he or she needed time to think or additional information, whether he or she asked for information, from whom...

#### Practice of ADVIH

Acceptance / refusal to do ADVIH? What is the reason for this?

Practice of self-testing: have them describe where and how the TA took place, with the partner or alone...

Ease / difficulty of use? Ease / difficulty in understanding the test result?

Test result: information sharing with the partner? With other people?

Perception of test results: reliability, doubt?

Approach implemented after the TA, depending on the result. If the result is positive: medical consultation (alone or accompanied by the partner?), implementation of a follow-up, treatment...

#### Impacts of ADVIH

On the relationship: trust, communication, distance, violence, sexuality...

On access and relationship to care (especially if the result is positive)

On testing practices, prevention practices, the relationship to HIV, to risk...

## 3. Thematic interview guide - Healthcare workers

#### General information

Age

Vocational training

Workplace

Professional activity

Involvement in the care of people living with HIV/AIDS

Description of the service's activities

Active file of PLWHIV

#### ADVIH and screening of partners of PLWHAs

Before the introduction of ADVIH: position/activities of the service in relation to the screening of PLWHIV partners, support for PLWHIV in sharing their status with the partner.

Date and progress of the implementation of ADVIH for PLWHA partners: inconveniences, obstacles, tools mobilised to implement ADVIH for PLWHA partners, adaptation of consultations, information mobilised, choice of PLWHA to whom ADVIH is proposed for their partner...

Confidentiality management

#### Attitudes of PLWHIV towards ADVIH's proposal for their partners

Content of the information presented to them (describe the terms used to present ADVIH to them)

Refusal / acceptance / doubt / questions

#### Attitudes of PLWHIV and/or partners after use of ADVIH

Responses of PLWHIV and partners after testing based on the results (do they inform about the results? How? (By telephone; face-to-face; accompanied; indirect information via partner) time frame)

#### Support for partners with a positive ADVIH result

Confirmation of status after TA screening

Role of the PLWHIV in the support of its partner

Care-giver-care relationship when screening was done by ADVIH

## 4. Thematic interview guide - Field return module

#### PvHIV

Looking back: advantages/disadvantages of ADVIH

Longer-term impacts on relationship, care, sexuality, HIV communication

Impacts on partner screening practices (repeated practice of ADVIH...)

What had not been anticipated (positive points, negative points)?

At the time of the proposal: sufficient information on ADVIH?

How can this screening strategy be improved?

#### Partner of PvVIH

Looking back: advantages/disadvantages of ADVIH

Longer-term impacts on relationship, care, sexuality, HIV communication

Repeated practice of ADVIH? Or other modes of screening?

At the time of the proposal: sufficient information on ADVIH?

How can this screening strategy be improved?

#### Health person

Looking back: advantages/disadvantages of ADVIH for partners

Positive / negative / expected / unexpected impacts

How can this screening strategy be improved?

**WP Survey Coupons**

WP Survey Coupons

Information leaflet (website)

# ATLAS: HIV Self-Test, Free Access to Know Your HIV Status

Version 2.1 of 5 August 2019 approved by the WHO Research Ethics Committee (date) and the Ethics Committees of Côte d'Ivoire (opinion, date), Mali (opinion, date) and Senegal (opinion, date).

**Contact**: XXX

**Principal Investigator** : Joseph Larmarange, CEPED/IRD, Paris, France. Contact: joseph.larmarange@ird.fr

**Responsible for data processing**: Institut de Recherche pour le Developpement, 44 bd de Dunkerque, Marseill, France. Contact: dpo@ird.fr

**Financeur** : Unitaid, Global Health Campus, Chemin du Pommier 40, 5th floor, 1218 Grand-Saconnex, Geneva, Switzerland. Tel. +41 22 791 12 00

## Research in the context of the ATLAS programme

Following the recommendations of the World Health Organization and the experience gained in East and Southern Africa, Unitaid wanted to promote and deploy HIV self-tests in West Africa through the funding of the ATLAS project in Côte d'Ivoire, Mali and Senegal, led by a consortium composed of the NGO Solthis (lead partner) and the Institut de Recherche pour le Développement (IRD) for the research part.

One of the objectives of the ATLAS programme is to assess the different channels of distribution of HIV self-test kits.

## Procedures

You have been selected because you used an HIV self-test and called the toll-free number on the package.

This research consists of an individual interview that will last approximately 30 minutes. If you agree to participate, you will be asked questions about your knowledge and opinions about the self-test you have carried out. For reasons of follow-up to our survey, our interview will be recorded, anonymously, if you agree. The recordings will be destroyed at the latest at the end of the project.

No one outside the research team will have access to the information you are about to give us. All files will be kept confidential. Your name will never be collected.

Only if you agree, we will ask you for your telephone number to call you back for an interview or a complementary questionnaire. Your telephone number will be destroyed at the end of the survey (by autumn 2021 at the latest) and your answers will remain confidential. Once your telephone number has been destroyed, your answers will be anonymous.

## Your participation in this study is entirely voluntary

You are free to withdraw or not answer the questions at any time you wish. There will be no consequences if you decide not to participate or if you decide to withdraw before the end of the discussion.

## What are the risks you face if you participate in this study?

In our opinion, there are no major risks associated with participating in this study. The only risk we see in this study is social, i.e. someone outside the study may find out about your status or practices/behaviours through participation in this study. But we minimise this risk by not collecting your name.

## What are the benefits of participating in this study?

## There are no individual benefits to participating in this study, but your participation will give us a better understanding of how to organise the provision of HIV self-testing and thus participate in the implementation of the provision of HIV self-testing in your country.

## Compensation

## You will not be paid for this study. The call is free of charge.

## Data processing

The data collected is the data you will give us during the interview. You are free not to answer one or more questions. Your data will be analysed in relation to the objectives of this research. You have a right of access, a right to rectify your collected data, the right to object (right of opposition) and the right to limit their use (right to limit processing). These rights are exercised with the investigator or the principal investigator of the study during the interview. If you have provided your telephone number, you will be able to exercise your right of access until the end of the project (end 2021). After this date, your telephone number will be deleted from our database and your data will be anonymous. Finally, your personal data will be kept for the time necessary for their analysis and publication of the results (maximum 5 years after the end of the project) and then your anonymised data will be archived in accordance with the regulations in force for a long period of time.

## If you have any questions

If you have any doubts or if you need further clarification, you can contact the mediator of this study at this number: tel. no. __XXXXXXXXXXXX (enquiry line number)

## Your rights as a participant

Again, your participation is voluntary and I repeat that you are free to change your mind at any time regarding your participation or to refuse to answer certain questions. This study has been reviewed and approved by the Ethics Committees of Cote d'Ivoire, Mali and Senegal, and the WHO Ethics Committee.

A website dedicated to the ATLAS programme has been opened [(](https://www.google.com/url?q=https://atlas.solthis.org/&sa=D&ust=1552897359532000&usg=AFQjCNFeeDHuOImRkHc0ycsy0zeHGUG_fQ)https://atlas.solthis.org/). You will be able to find this information notice as well as the research results at the end of the project.

If you have any questions about the way you are treated in the study or your rights as a participant, you can contact the Ethics Committee that has agreed to the study in your country at the following address__________________________________________________________________XXXXXXXXXXXXXXXXXXXXXXXXXXXXXXXXXX

At the end of this study, you will be able to find out the results from XXX (local NGO?).

**Date on which the information was issued:** I__I__I / I__I__I / I__I__I

**By:** (surname, first name, position)


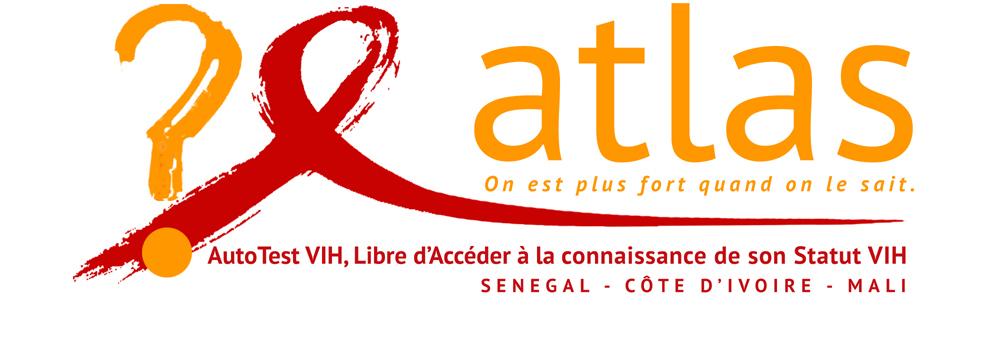


Questionnaires survey Coupons

Version 2.1 of August 5, 2019

# General instructions

- unless otherwise specified, cite all possible ways of responding to the respondent
- unless otherwise specified, only one possible answer per question
- when there is an "other, specify" mode, provide a textual variable for entry

*This questionnaire will need to be tested with a small sample before the survey is carried out.*

# Long questionnaire phase 1

CONTACT, CONSENT AND ELIGIBILITY PHASE

**Introductory text and presentation of the survey**

Good morning Madam / Sir

First of all, I would like to thank you for calling the << toll-free number>>.

It is in the context of improving HIV-AIDS testing policies and access to care in Sub-Saharan Africa that the NGO Solidarité Thérapeutique et Initiative de Santé (SOLTHIS) and the Institut de Recherche et de Développement (IRD), in partnership with the Ministry of Health, undertook to carry out this public health study. It is being carried out in three countries: Senegal, Côte d'Ivoire and Mali.

If we want to improve HIV-AIDS prevention and testing, it is important to collect information from people who have already used HIV self-testing.

*Note: in all cases, if the person needs advice or support, please refer to the national information line and offer to call us back or make an appointment.*

## Consent

**INTRO.01** Would you agree to give us 15 minutes of your time to answer our anonymous questionnaire? (We are not going to ask for your first or last name)

1. Yes, immediately
2. Yes, but later=> making an appointment
3. No

You have the right to

**INTRO.02** If not, why do you refuse to participate?

1. I just wanted to get information about AIDS and/or self-tests.
2. I don't like being asked questions on the phone
3. Other to be specified

*Note: in all cases, if someone needs advice or support, please refer to the national information line and offer to call us back or make an appointment.*

**INTRO.03**. Country (collected automatically from the toll-free reception number)

1. Senegal
2. Ivory Coast
3. Mali

**INTROCONSENT**. If INTRO.01=1, we thank you for your participation in this study and for your time. We would like to remind you that this questionnaire is strictly anonymous and confidential**.**

**CONSENTAUDIO.** For the purposes of following up our survey, our interview will be recorded, anonymously, if you agree. The recordings will be destroyed at the end of the project.

**INTRO.04** Would you agree to have our interview recorded?

1. Agree to registration
2. No agreement for registration

## Eligibility

**El1.in** order to check if you are eligible to participate in the study, can you tell me your age?

|____| age. (min=0, max=99)+ Don't know

If age= 15 or less in Côte d'Ivoire, 17 or less in Mali, 14 or less in Senegal → not eligible FIN

**El1Bis**. If El1=NSP, what is your date of birth?

|__|__|__|__||__| Ann |__|__|__| Months

+ (Don't know)

**EL1TER.** If EL1Bis=NSP, Age recoded

|____| years

if 14 years old or younger and country=Senegal → FIN questionnaire

if 15 years or less and country=Côte d'Ivoire → FIN questionnaire

if 17 years old or younger and country=Mali → FIN questionnaire

98. Refusal => End of questionnaire

99. Don't know => end of questionnaire

**EL2.** Have you used or tried to use an HIV self-test kit?

1. Yes => SD01
2. No

**EL2Bis**. If EL2=no, we invite you to use the HIV self-test kit, and call us back later, or offer us an appointment in a few days.

1. Ok, I'll call you back later => End of questionnaire
2. Ok, I'll make an appointment

**EL2TER**. Date and time you wish to be called back.

|__|__|__|__||_2_|_0_|__|__|__|__|__|__|__|__|__||__| => End of questionnaire

(Da y) (month) (hour) (minute) (second)

## Socio-demographic characteristics

**SD01**. What is your gender? (Ask only in case of doubt, otherwise fill in directly)

1. Man
2. Woman

**SD02**. What is your marital status?

1. Single (Never married)
2. In a common-law relationship
3. Married (monogamous, polygamous)
4. Divorced/separated
5. Widow.Widower

**SD03**. Can you read or write?

1. Yes, perfectly
2. Yes, moderately
3. No, not at all

**SD03Bis**. What is the highest level of study you have achieved?

1. Did not attend school
2. Koranic school only
3. Primary
4. Secondary (middle school, general or vocational school)
5. Higher education (University, grande école...)

**SD04**. In which region do you live?

*(list of regions by country pre-coded)*

**SD05.** Financially speaking, would you say ?

1. You are comfortable
2. Your income is enough for you
3. You are poor
4. You are very poor

**SD06**. Compared to people of your age, would you say that your state of health is?

1. Not at all satisfactory
2. Unsatisfactory
3. Quite satisfactory
4. Very satisfactory
5. Don't know

## Screening history

**HD01.** In the past (excluding the self-test you just did), have you ever had an HIV test? (Regardless of the type of test)

1. Yes
2. No

**HD02**. If yes, how many times had you had a screening test? *(Try to get an estimate in case the respondent does not remember the exact number of tests already carried out.)*

|____|||tests (min=0,max=99)

**HD02Bis**. If not, why have you never done it? (Do not quote)

1. I was afraid that my status would be known by other people if I was declared positive.
2. I didn't know where to get tested/ Didn't know who to go to
3. Screening sites were too far away / Transportation costs are high
4. I didn't have the time
5. Tests are not reliable
6. I was afraid of the result
7. I don't think I'm infected / I never take risks
8. Other to be specified

**HD03**. How long ago was the last time you took the test?

1. Less than 1 month
2. Between 1 and 3 months
3. Between 3 and 6 months
4. More than 6 months
5. More than 12 months
6. More than 2 years
7. More than 5 years

**HD03Bis**. When did you take this test?

1. On the occasion of a prenatal consultation
2. During a consultation for a health problem (STI, malaria...)
3. On the occasion of a health check-up
4. On the occasion of a blood donation
5. Following risk-taking / condom breakage
6. I wanted to stop using condoms with my partner.
7. Following an awareness-raising campaign
8. You had learned that your partner/one of your partners was infected with HIV.
9. Other to be specified

**HD04**. Where did you do it?

1. In a voluntary testing centre (VCT outside a health centre)
2. In the maternity ward / antenatal clinic
3. In a health centre outside the prenatal service (CHU, CHR, CSU, CSR...)
4. As part of a mobile screening campaign
5. In a blood transfusion centre
6. At the workplace or company / At school / University
7. With a self-test
8. Other to be specified

**HD05**. Did you know the result?

1. Yes
2. No

**HD05Bis**. If not, why not? (Do not quote)

1. I was afraid to know the result
2. Waiting time for the result was too long
3. I was afraid that my status would be known to others, if I was infected.e
4. I was unable to read my result
5. Other to be specified

**HD06**. What was the result? (You don't have to answer me)

1. Negative
2. Positive
3. Undetermined
4. Refuses to answer

## Experience of ADVIH

**EXP01.** Do you have the self-screening kit nearby or can you go and get it.

1. Yes
2. No

**EXP02.** IF yes, for statistical reasons, could you provide us with the colour and three-digit code of the round sticker glued to the brochure accompanying the self-screening kit, right next to the invitation to participate in the survey?

Number code :

Colour :

**EXP03.** How did you retrieve this self-test? Who gave you this self-test?

1. A health professional
2. A peer educator / community health worker
3. One / One sexual partner
4. A / A friend
5. One / One colleague
6. One / One acquaintance
7. Other, please specify

**EXP04**. Is your self-test an oral test or a blood test?

1. Oral test
2. Blood test
3. Don't know

**EXP05**. Did you have difficulty understanding the instructions for use?

1. Yes
2. No

**EXP05BIS**. If yes, explain.

| |______________________________________________________

**EXP06**. Did you receive any help or information before or during the completion of your self-test via the following means? (ask yes / no)

(If EXP06==1)

1. Health professional,
2. Community worker,
3. Partner,
4. Friend/acquaintance
5. Hotline / Toll-free number / AIDS Info Line (*adapt name to each country)*
6. Information leaflets / Documentation supplied with the self-test
7. Demonstration videos (on the internet or not)
8. Other to be specified

**EXP07**. Did you have any difficulties in performing the sample collection?

*(adapt wording according to EXP04)*

1. Yes
2. No

**EXP07BIS**. If yes, please give details.

| |______________________________________________________

**EXP08.** How long did you wait before reading the result? (Do not quote modalities)

1. Less than 20 minutes
2. Between 20 and 40 mn
3. More than 40 mn
4. Don't know

**EXP09.** Did you have difficulty reading the result?

1. Yes
2. No

**EXP09BIS**. If yes, explain.

| |______________________________________________________

## Result of the self-test

**EXP10.** What was the result of your self-test? (I remind you that you are not obliged to answer me)

1. Non-reactive test (negative)
2. Reagent test (positive)
3. Not valid
4. Refuses to answer
5. DK (did not know how to interpret the result)

**EXP11**. How many lines were visible when reading the result of your self-test? (if EXP04==1)

1. No lines
2. 1 Line
3. 2 lines

*Note: if the person always has the kit with him/her, invite him/her to read the result directly.*

*Note: this question will be adapted once blood tests are introduced in the countries.*

**EXP11BIS**. Was this line the top or the bottom one? (to be put down only if EXP11=2)

1. The bottom line
2. The top line
3. Don't know

**EXP12**. Before you received this HIV self-test kit, had you ever heard about it in the following media? *(multiple choice, ask yes/no)*

1. Health professional
2. Community worker/association
3. Sexual or sentimental partner
4. Friend/acquaintance
5. Television
6. Radio/newspapers
7. Internet
8. Other to be specified

**EXP13**. Have you ever heard of or followed the TV, radio and internet series "MTV Shuga BABI"?

1. Yes
2. No
3. Don't know

## Satisfaction

**SAT01.** Would you say the use of self-testing for HIV is :

1. Very simple
2. Simple
3. Not simple
4. Not at all simple

**SAT02**. Would you say that reading the result of the HIV self-test is :

1. Very easy
2. Easy
3. Not easy
4. Not at all easy

**SAT03**. After using the HIV self-test, would you say that you are :

1. Totally satisfied
2. Partially satisfied
3. Not satisfied
4. Not at all satisfied

**SAT04.** What did you like about self-screening? *(ask each modality in yes/no/NSP)*

1. Ease of use
2. Discretion / anonymity
3. Reliability of the result
4. Autonomy in carrying out screening
5. Free test

Sexual behaviour

**CP01:** Have you ever had sexual intercourse in your life?

*(Sexual intercourse is defined here as any penetration of the vagina by a penis or any penetration of the anus by a penis.)*

1. Yes
2. No

**CP02.During** your life, have you had sexual relations with?

1. Only with men
2. Only with women
3. With men and women
4. Refuses to answer

**CP02.01**. How many male sexual partners have you had sex with in the last 12 months? (ask if CP02==1 or==3)

|__|______ men. (min=0, max=999)

**CP02.02**. How many female sexual partners have you had sex with in the last 12 months? (ask only if CP02==2 or ==3)

|__|____| women. (min=0, max=999)

of which question on prostitution (client / TS)

**CP06**. Have you ever received money in exchange for sex in your life?

1. Yes, often
2. Yes, occasionally
3. No, never
4. Refuses to answer

**CP06Bis**. When was the last time you did this?

|____| Years

|__________ Month

**CP07**. Have you ever been given money in exchange for sex in your life?

1. Yes, quite often
2. Yes, occasionally
3. No, never
4. Refuses to answer

**CP07Bis**. How long ago was the last time you did it?

|____| Years

|__________ Month

**CP08**. Have you ever been a prostitute at least once in your life?

1. Yes, quite often
2. Yes, occasionally
3. No, never
4. Refuses to answer

**CP08BIS**. When was the last time you were involved in prostitution? (if CP08==1 or 2)

|____| Years

|__________ Month

**CP09.** Have you ever been a client of a prostitute?

1. Yes, quite often
2. Yes, occasionally
3. No, never
4. Refuses to answer

**CP09BIS. When was the** last time you visited a prostitute?

|____| Years

|__________ Month

**CP10**. In the past 12 months, with your sexual partners (regular and casual), have you used a condom?

1. Always
2. Occasionally
3. Never
4. Refuses to answer

**CP11**. Do you think that you are very, somewhat or not at all exposed to the risk of catching the AIDS virus?

1. Very exposed
2. A little exposed
3. Not at all exposed
4. Don't know

## Recontact

*IF test is reactive or indeterminate or does not over-interpret the result:*

**REC01** We would like to contact you again in three months for a second part of the survey. It will be a short questionnaire and, as with this one, your answers will remain anonymous. Do you agree to be contacted by telephone in three months' time? If you say yes, you will always have the possibility to refuse when we contact you again.

1. Yes
2. No

*ALL*

**REC02**. Also as part of this research, we are looking for people who have used a self-test and who would be interested in participating in another survey. This would be an open-ended telephone discussion of one to one and a half hours with an interviewer. Your anonymity would be preserved. Would you be interested in participating in this qualitative component? If you say yes, you can always refuse later, if we contact you again. Furthermore, you will not necessarily be contacted again as this survey would only involve a small number of people.

1. Yes
2. No

If yes to REC01 or REC02

**REC03**. On which number(s) would you like to be contacted? (two contacts at least)

|__|__||__|__||__|__||__|__|

|__|__||__|__||__|__||__|__|

|__|__||__|__||__|__||__|__|

(if country= Senegal)

**REC04.** Do you have a first name or nickname that you would be willing to give us to make sure that it is you who picks up the phone when we call you back?

| |____________________________________

## Acknowledgements

End text

**This was my last question, thank you for your time.**

**If you have any questions about HIV, HIV testing or the medical management of HIV, do not hesitate to contact a national helpline: the** Info Sida 106 line in Côte d'Ivoire, the Kènèyako line 80 00 11 81 / 80 00 28 28 in Mali and the CTA Info Santé line 0800 00 30 30 in Senegal.

**I wish you an excellent (end) day.**

*Interviewer, indicate the language in which the questionnaire is to be completed.*

**LG.** Language of the questionnaire

| |____________________________________

#


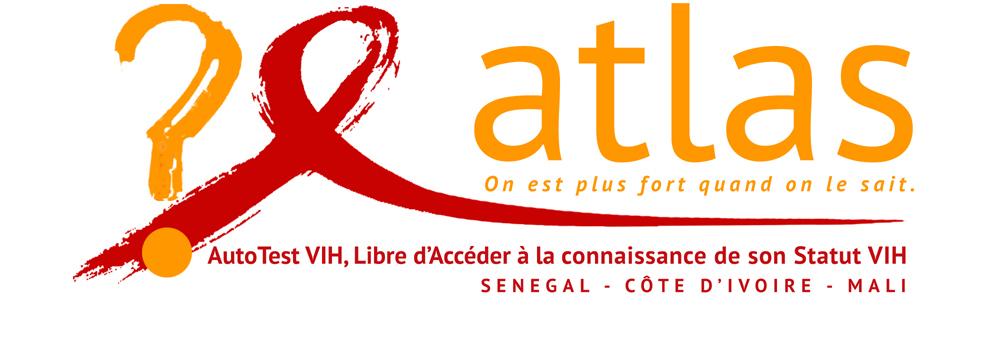


Questionnaires survey Coupons

# Short questionnaire phase 1

Version 2.1 of August 5, 2019

CONTACT, CONSENT AND ELIGIBILITY PHASE

**Introductory text and presentation of the survey**

Good morning Mrs / Mr

First of all, I would like to thank you for calling the << toll-free number>>.

It is in the context of improving HIV-AIDS testing policies and access to care in Sub-Saharan Africa that the NGO Solidarité Thérapeutique et Initiative de Santé (SOLTHIS) and the Institut de Recherche et de Développement (IRD), in partnership with the Ministry of Health, undertook to carry out this public health study. It is being carried out in three countries: Senegal, Côte d'Ivoire and Mali.

If we want to improve HIV-AIDS prevention and testing, it is important to collect information from people who have already taken HIV self-tests.

*Note: in all cases, if the person needs advice or support, please refer to the national information line and offer to call us back or make an appointment.*

## Consent

**INTRO.01** Would you agree to give us 3 minutes of your time to answer our anonymous questionnaire (we are not going to ask for your first or last name)?

1. Yes, immediately
2. Yes, but later=> making an appointment
3. No

**INTRO.02** If not, why do you refuse to participate?

1. I just wanted to get information about AIDS and self-testing.
2. I don't like being asked questions on the phone
3. Other to be specified

*Note: in all cases, if someone needs advice or support, please refer to the national information line and offer to call us back or make an appointment.*

**INTRO.03**. Country (collected automatically from the toll-free reception number)

1. Senegal
2. Ivory Coast
3. Mali

**INTRO.04**. If INTRO.01=1, we thank you for your participation in this study and for your time. We would like to remind you that this questionnaire is strictly anonymous and confidential (we are not going to ask for your first or last name).

**CONSENTAUDIO.** For survey monitoring purposes, your interview may be recorded, anonymously, unless you object. Do you agree?

1. Agree to registration
2. No agreement for registration

## Eligibility

**El1.in** order to check if you are eligible to participate in the study, can you tell me your age?

|____| age. (min=0, max=99)+ Don't know

If age= 15 or less in Côte d'Ivoire, 17 or less in Mali, 14 or less in Senegal → not eligible FIN

**El1Bis**. If El1=NSP, what is your date of birth?

|__|__|__|__||__| [1900 - 2004] + (Don't know)

**EL1TER.** If EL1Bis=NSP, Age recoded

|____| years

if 14 years old or younger and country=Senegal → FIN questionnaire

if 15 years or less and country=Côte d'Ivoire → FIN questionnaire

if 17 years old or younger and country=Mali → FIN questionnaire

98. Refusal => End of questionnaire

99. Don't know => end of questionnaire

**EL2.** Have you used or tried to use an HIV self-test kit?

1. Yes => SD01
2. No

**EL2Bis**. If EL2=no, we invite you to use the HIV self-test kit, and call us back later, or offer us an appointment in a few days.

1. Ok, I'll call you back later => End of questionnaire
2. Ok, I'll make an appointment

**EL2TER**. Date and time you wish to be called back.

|__|__|__|__||_2_|_0_|__|__|__|__|__|__|__|__|__||__| => End of questionnaire

(Da y) (month) (hour) (minute) (second)

## Socio-demographic characteristics

**SD01**. What is your gender? (Ask only in case of doubt, otherwise fill in directly)

1. male

2. Woman

**SD03.** Can you read or write?

1. Yes, perfectly
2. Yes, moderately
3. No, not at all

**SD03BIS**. What is the highest level of study you have achieved?

1. Did not attend school
2. Koranic school only
3. Primary
4. Secondary (middle school, general or vocational school)
5. Higher education (University, grande école...)

**SD04**. In which region do you live?

## Test result

**EXP01.** Do you have the self-screening kit nearby or can you go and get it.

1. Yes
2. No

**EXP02.** IF yes, for statistical reasons, could you provide us with the colour and three-digit code of the round sticker glued to the brochure accompanying the self-screening kit, right next to the invitation to participate in the survey?

**Number code** :

**Colour** :

**EXP04**. Is your self-test an oral test or a blood test?

1. Oral test
2. Blood test
3. Don't know

**EXP10.** What was the result provided by your self-test? (I remind you that you are not obliged to answer me)

1. Non-reactive test (negative)
2. Reagent test (positive)
3. Not valid
4. Refuses to answer
5. DK (did not know how to interpret the result)

**EXP11**. How many lines were visible when reading the result of your self-test? (to be asked only if EXP04=1)

1. No lines
2. 1 Line
3. 2 lines

*Note: if the person always has the kit with him/her, invite him/her to read the result directly.*

*Note: this question will be adapted once blood tests are introduced in the countries.*

**EXP11BIS**. Was this line the top or the bottom one? (to be put down only if EXP11=2)

1. The bottom line
2. The top line
3. Don't know

## Recontact

*IF test is reactive or indeterminate or does not over-interpret the result:*

**REC01** We would like to contact you again in three months for a second part of the survey. It will be a short questionnaire and, as with this one, your answers will remain anonymous. Do you agree to be contacted by telephone in three months' time? If you say yes, you will always have the possibility to refuse when we contact you again.

1. Yes
2. No

*ALL*

**REC02**. Also as part of this research, we are looking for people who have used a self-test and who would be interested in participating in another survey. This would be an open-ended telephone discussion of one to one and a half hours with an interviewer. Your anonymity would be preserved. Would you be interested in participating in this qualitative component? If you say yes, you can always refuse later, if we contact you again. Furthermore, you will not necessarily be contacted again as this survey would only involve a small number of people.

1. Yes
2. No

(If yes to REC01 or REC02)

**REC03**. On which number would you like to be contacted? (two contacts if possible)

(if country= Ivory Coast or Mali)

|__|__||__|__||__|__||__|__|

|__|__||__|__||__|__||__|__|

|__|__||__|__||__|__||__|__|

(if country= Senegal)

**REC04.** Do you have a first name or nickname that you would be willing to give us to make sure that it is you who picks up the phone when we call you back?

| |____________________________________

## Acknowledgements

End text

**This was my last question, thank you for your time. If you have any questions about HIV, HIV testing or medical care for HIV, do not hesitate to contact a national helpline: the** Info Sida 106 line in Ivory Coast, the Kènèyako 80 00 11 81 / 80 00 28 28 line in Mali and the CTA Info Santé 0800 00 30 30 line in Senegal.

**I wish you an excellent (end) day.**

*Interviewer, indicate the language in which the questionnaire is to be completed.*

**LG.** Language of the questionnaire

| |____________________________________


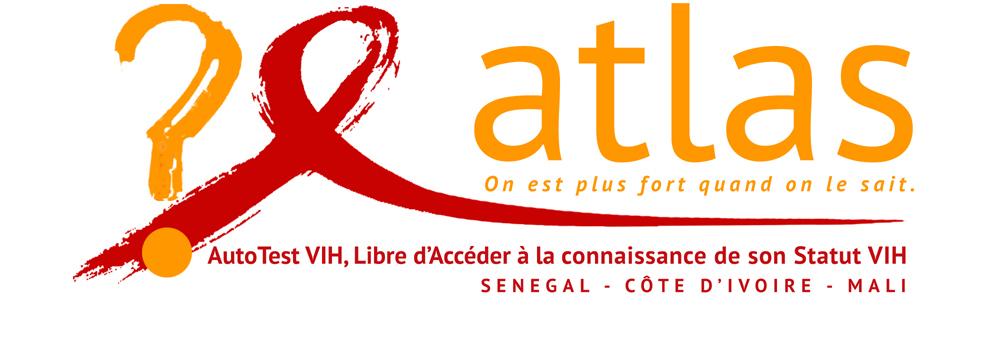


Questionnaires survey Coupons

# Questionnaire phase 2

Version 2.1 of August 5, 2019

**Introductory text**

Good morning Mrs / Mr

I have been mandated by the NGO Solidarité Thérapeutique et Initiative de Santé (SOLTHIS), the Institut de Recherche et de Développement (IRD) and the Ministries of Health who are carrying out phase 2 of their public health study, of which you participated in phase 1 three months ago and at that time you gave your contact details in order to take part in this second interview.

We would like to ask you a few questions about your care path. This could take you about 5 minutes.

## Consent

**Consent01**. Do you agree to participate (I remind you that this questionnaire is anonymous and strictly confidential)

1. Yes, immediately
2. Yes, making an appointment
3. No, refusal

**ConsenRef**. why do you refuse to participate?

| |=>End_______________________________________________________________ questionnaire

**CONSENTAUDIO.** For reasons of survey follow-up, it is possible that your interview will be recorded anonymously, unless you object. Do you agree?

1. Agree to registration
2. No agreement for registration

## Link to care

**PS01.** Following the HIV self-test you did three months ago, did you consult a health professional for a conventional HIV test called a confirmatory test?

1. Yes
2. No

**PS01BIS.** Since the HIV self-test you did about 3 months ago, whose result you were unable to interpret, have you done another HIV test, either with a health professional, a community health worker or in a laboratory? (if EXP10==5 in phase 1)

1. Yes
2. No

**PS02.** If not, why not?

| |___________________________________________

(if PS01=1)

**PS03.** How long after the self-test? (if PS01==1 or PS01BIS==1)

1. Less than a week after the self-test
2. Between 1 and 2 weeks
3. Between 2 and 4 weeks
4. Between 1 and 2 months after
5. Between 2 and 3 months after

**PS04.** Where did you do this test? (place of the confirmatory or invalidation test)

1. Voluntary testing centre (VCT) outside the health centre
2. Maternity / Prenatal clinic
3. Health Centre / Hospital / Clinic
4. Community Clinic / Association
5. Dedicated health centre (HS / TS / UD)
6. Analysis laboratory
7. Other to be specified

**PS05**. What was the result?

1. Positive
2. Negative
3. Refusal
4. NSP

(If confirmatory test is positive) :

**PS06**. Have you seen a health professional to get your HIV infection managed?

1. Yes
2. No

**PS07**. If yes, how long after the confirmatory test?

1. Less than a week after the self-test
2. Between 1 and 2 weeks
3. Between 2 and 4 weeks
4. Between 1 and 2 months after
5. Between 2 and 3 months after

**PS07BIS**. If not, why not?

| |_______________________________________________

**PS08** Did they tell you about HIV treatment that keeps you healthy?

1. Yes
2. No
3. Don't know

**PS09**.if yes, did they prescribe this treatment?

1. Yes
2. No

**PS10.** If yes, have you started taking this treatment?

1. Yes
2. No

**PS10BIS**. If not, why not?

| |_______________________________________________

**PS11**. If yes, are you still taking this treatment today?

1. Yes
2. No

**PS11BIS**. If not, why not?

| |_______________________________________________

**PS12.** Do you know if you have had a medical examination called CD4 count? (if PS06==1)

1. Yes
2. No
3. NSP

**PS12BIS**. IF yes, do you know/remember the result?

|__|__|__||__| CD4

NSP

## Text conclusion

**This was my last question, thank you for your time.**

**If you have any questions about HIV, HIV testing or the medical management of HIV, do not hesitate to contact a national helpline: the** Info Sida 106 line in Côte d'Ivoire, the Kènèyako line 80 00 11 81 / 80 00 28 28 in Mali and the CTA Info Santé line 0800 00 30 30 in Senegal.

**I wish you an excellent (end) day.**

*Interviewer, indicate the language in which the questionnaire is to be completed.*

**LG.** Language of the questionnaire

| |____________________________________

**WP Economic Component**

WP Economic Component

Information leaflet on cost studies

Version 2.1 of 5 August 2019 approved by the WHO Research Ethics Committee (date) and the Ethics Committees of Côte d'Ivoire (opinion, date), Mali (opinion, date) and Senegal (opinion, date).

**Principal Investigator** : Fern Terris-Persholt, CEPED/IRD, Paris, France. Contact: joseph.larmarange@ird.fr

### Information on the project

We are health economists working for the NGO Solthis and at the London School of Hygiene and Tropical Medicine (LSHTM). We conduct HIV research for your country and region.

Regular HIV testing is very important in your country and around the world because it helps HIV-positive people get treatment while they are still healthy and can also help reduce the spread of HIV. Self-testing for HIV is an innovative way to test yourself and is being introduced in your country.

ATLAS is a research project that we hope will help us understand how best to introduce HIV self-testing in your country. The study we are conducting is part of the ATLAS project. It is an analysis of the costs of conventional HIV testing, and the costs associated with providing HIV self-testing.

1. *Interview with the director of the health service (or other competent authority)*

During this interview, we will ask you questions about how your facility operates for the provision of HIV care services, in order to identify and estimate the costs associated with conventional HIV testing and HIV self-testing. We will ask you to provide us with financial data.

1. *Study of time and movement with HIV care providers*

The purpose of this study is to estimate the amount of time it takes care providers to provide HIV testing services and HIV self-testing to a patient. This will allow us to estimate the staff costs directly associated with providing HIV care by estimating their time spent on this activity.

This involves sessions to time HIV testing visits and/or provide HIV self-screening by HIV care providers. Caregivers participating in the study will be asked to sign a consent form. Observations will be made over a full working day.

This can be either for an HIV testing service based in a health facility or mobile, or for a self-test that the patient will perform himself/herself, or that he/she might suggest to a partner.

We would like to reassure participants that the aim of this study is not to "control" the quality of care provided, but only to better understand the time it takes to carry out HIV tests/self-tests.

The researcher will be present during the consultation with the agreement of the patient. If the patient wishes to be alone with the caregiver, the researcher will remain outside the consultation room.

### Confidentiality of study data

All information from the study will be stored securely in paper and computer files, and only researchers participating in the study will have access to it. We will use a number to identify the participant. For the study of time and motion, we will not record any names, only professional status will be recorded. The data provided will be analysed, and confidentiality will be maintained throughout the data processing and storage processes.

The data we collect may be published in scientific journals and project reports so that others can learn from your experience. The data may also be made available to other researchers so that it can be used to improve the delivery of HIV-related services.

### Approval by research ethics committees

The study has been reviewed and approved by your country's national ethics committee, the World Health Organization and the London School of Hygiene and Tropical Medicine.

If you have any questions about this study, please do not hesitate to ask them now. If you have any questions after we have left, please do not hesitate to contact us by calling the following number and ask for (Surname, First name) (principal researcher): Tel: XXX.

### Data processing

The data collected is the data you will give us during the interview. You are free not to answer one or more questions. Your data will be processed in order to analyse the results of the research with regard to its objectives.

You have a right of access, a right to rectify your collected data, the right to object (right of opposition) and the right to limit their use (right to limit processing). These rights can be exercised by contacting the interviewer or the principal investigator of the study by giving your interview number. Finally, your personal data will be kept for the time necessary for their analysis and the publication of the results (maximum 5 years after the end of the project) and then your anonymised data will be archived in accordance with the regulations in force for a long period of time.

### More information on the ATLAS programme

A website dedicated to the ATLAS programme has been opened [(](https://www.google.com/url?q=https://atlas.solthis.org/&sa=D&ust=1552897359532000&usg=AFQjCNFeeDHuOImRkHc0ycsy0zeHGUG_fQ)https://atlas.solthis.org/). You will be able to find this information notice as well as the research results at the end of the project.

## Interview with the director of the health centre (or other competent authority) for the provision of costs associated with the provision of HIV testing services

## –

## Consent Form

The information leaflet, Version 2.1 of 5 August 2019, describing the purpose, methods, benefits and risks of research on the costs (individual interviews) of HIV self-testing was read and explained to me. I had the opportunity to have satisfactory answers to all the questions about the study and had enough time to think about my participation.

If you would like more information, please do not hesitate to ask us questions, read the information brochure provided.

Do you have any questions about the study?

Do you agree to take part in this interview?

□ I agree to be registered. □ I do not agree to be registered.

If you have no further questions and agree to participate in the study, please sign this form, indicating that I have informed you of your rights as a participant and that you have agreed to participate in the research. Thank you for your time.

PARTICIPANT] I have read the information in the fact sheet for this study (or it was read to me). All my questions about the study and my participation in it have been answered. I freely agree to participate in this study.

_____________________________________________________________________________

Name of participant (in capital letters) Signature Date

_____________________________________________________________________________

Researcher's name (in capital letters) Signature Date

**Economic analysis of the integration of HIV Self-Testing (HIV SIT) into conventional HIV testing in Côte d'Ivoire, Mali and Senegal**

**Periodic review of the activities of institutions offering**

**Integrated HIV testing services**

**Thematic guide for information collection**

**-**

**London School of Hygiene and Tropical Medicine**

**-**

**THIS GUIDE WILL SERVE AS A BASIS FOR ECONOMISTS TO DEVELOP A DATA COLLECTION FORM ADAPTED TO THE HIV/AIDS DELIVERY CHANNELS PER COUNTRY. THIS FORM WILL THEN BE TESTED FOR VALIDATION.**

**Name of the health centre:**

**Contact person :**

**Date of interview :**

**Period of data collection (target: 12 months prior to the date of the interview):**

**……………………………………………………………**

| **Section A: Environmental characteristics of the health centre** | | |
| --- | --- | --- |
| **Question** | **Answer** | **Comments** |
| Location of the health centre?   1. *Urban* 2. *Semi-urban* 3. *Rural* |  |  |
| How many ADVIH distributors work in this health centre? |  |  |
| How many health professionals work in the HIV department? |  |  |
| How many days a week does the health centre offer HIV testing services? |  |  |
| **General remarks** | | |

**COST OF CAPITAL**

**A. Infrastructure and stocks**

Data sources:

Annual rent cost or estimated value of the site (specify):

| **Spaces** | **Surface**  **(square meters)** | **% of use for HIV testing service provision** | **% of use for ADVIH service provision** |
| --- | --- | --- | --- |
|  |  |  |  |
| Entire site |  |  |  |
| Reception |  |  |  |
| Stock room |  |  |  |
| Laboratory |  |  |  |
| HIV testing room |  |  |  |
| Room ADVIH |  |  |  |
|  |  |  |  |
|  |  |  |  |
|  |  |  |  |
|  |  |  |  |
|  |  |  |  |
|  |  |  |  |

Furniture: can be estimated with + 10%.

**B. Equipment**

Data sources:

- What types of equipment are used for HIV testing services?
- Who paid for this equipment?
- What is the cost of each piece of equipment?
- How long does each piece of equipment last?

| **Equipment (list)** | **Quantity** | **Costs** | | **Service life** | **% allowance** |
| --- | --- | --- | --- | --- | --- |
|  |  | **The end.** | **Econ.** |  |  |
|  |  |  |  |  |  |
|  |  |  |  |  |  |
|  |  |  |  |  |  |
|  |  |  |  |  |  |
|  |  |  |  |  |  |
|  |  |  |  |  |  |
|  |  |  |  |  |  |
|  |  |  |  |  |  |
|  |  |  |  |  |  |
|  |  |  |  |  |  |
|  |  |  |  |  |  |
|  |  |  |  |  |  |
|  |  |  |  |  |  |
|  |  |  |  |  |  |
|  |  |  |  |  |  |
|  |  |  |  |  |  |
|  |  |  |  |  |  |
|  |  |  |  |  |  |
|  |  |  |  |  |  |
|  |  |  |  |  |  |

**Note:** use the current market price, not the purchase price.

**C. Vehicles**

Data sources:

- What types of vehicles are used?
- Who paid for each vehicle?
- What is the cost of each vehicle?
- How long does each vehicle last?

| **Vehicles (list)** | **Paid by** | **Costs** | | **Service life** | **% allowance** |
| --- | --- | --- | --- | --- | --- |
|  |  | **The end.** | **Econ.** |  |  |
|  |  |  |  |  |  |
|  |  |  |  |  |  |
|  |  |  |  |  |  |
|  |  |  |  |  |  |
|  |  |  |  |  |  |

**Notes:** use the current market price, not the purchase price.

**D. Other capital cost data (residual costs >$100)**

Data sources:

- What other capital costs does the programme include?
- Who paid for it?
- What is the cost of each product?
- How long does each product last?

| **Product (list)** | **Quantity** | **Costs** | | **Service life** | **% allowance** |
| --- | --- | --- | --- | --- | --- |
|  |  | **The end.** | **Econ.** |  |  |
|  |  |  |  |  |  |
|  |  |  |  |  |  |
|  |  |  |  |  |  |
|  |  |  |  |  |  |
|  |  |  |  |  |  |
|  |  |  |  |  |  |
|  |  |  |  |  |  |
|  |  |  |  |  |  |

**RECURRING COSTS**

**E. Staff**

Data sources:

- What categories of staff are involved in the programme (receptionist, HIV counsellor, nurse, doctor, health centre manager, volunteer, cleaning staff, etc.)?
- Who funds each category of staff?
- What are the gross annual salaries for each category?
- What are the annual costs of benefits received as employees?

| **Category of personnel** | **Quantity** | **Gross annual salary** | | **Annual profits received** | | **% allowance** |
| --- | --- | --- | --- | --- | --- | --- |
|  |  | **The end.** | **Econ.** | **The end.** | **Econ.** |  |
|  |  |  |  |  |  |  |
|  |  |  |  |  |  |  |
|  |  |  |  |  |  |  |
|  |  |  |  |  |  |  |
|  |  |  |  |  |  |  |
|  |  |  |  |  |  |  |
|  |  |  |  |  |  |  |

| **Position** | **Number of working days at the centre / week** | **% of time on the different activities** | | | | | |
| --- | --- | --- | --- | --- | --- | --- | --- |
|  |  | Reception | HIV and AIDS room | Supervision | ADVIH | Promotion | Others |
|  |  |  |  |  |  |  |  |
|  |  |  |  |  |  |  |  |
|  |  |  |  |  |  |  |  |
|  |  |  |  |  |  |  |  |
|  |  |  |  |  |  |  |  |
|  |  |  |  |  |  |  |  |
|  |  |  |  |  |  |  |  |
|  |  |  |  |  |  |  |  |
|  |  |  |  |  |  |  |  |
|  |  |  |  |  |  |  |  |
|  |  |  |  |  |  |  |  |

**Note:** Doctors, nurses, HIV counsellor, volunteers, receptionists, cleaning service, drivers, etc.

**F. Provisions**

Data sources:

- What provisions are used by the programme?
- Who pays for each provision?
- What are the annual quantities consumed (including waste and losses)?
- What is the unit cost of each product?

| **Product (list)** | **Annual quantity consumed** | **Costs** | | **% allowance** |
| --- | --- | --- | --- | --- |
|  |  | **The end.** | **Econ.** |  |
|  |  |  |  |  |
|  |  |  |  |  |
|  |  |  |  |  |
|  |  |  |  |  |
|  |  |  |  |  |
|  |  |  |  |  |
|  |  |  |  |  |
|  |  |  |  |  |
|  |  |  |  |  |

**Note:** Provision (equipment consumed in one year or <$100) - see guide at the end of the document. Should include supply costs and wastage.

**G. Costs associated with the operation of vehicles and transport**

Data sources:

- What are the costs over the observation period (e.g. petrol, oil, maintenance, insurance, registration, repairs, etc.)? )?
- Who pays for each expense?

| **Products (list)** | **Quantity** | **Costs** | | **% allowance** |
| --- | --- | --- | --- | --- |
|  |  | **The end.** | **Econ.** |  |
|  |  |  |  |  |
|  |  |  |  |  |
|  |  |  |  |  |
|  |  |  |  |  |
|  |  |  |  |  |
|  |  |  |  |  |
|  |  |  |  |  |
|  |  |  |  |  |

**H. Operation of the infrastructure**

| **Services** | **Phone** | **Water** | **Electricity** | **Maintenance/**  **repairs** | **Insurance** | **Other (specify)** |
| --- | --- | --- | --- | --- | --- | --- |
| **Payer** |  |  |  |  |  |  |
| January |  |  |  |  |  |  |
| February |  |  |  |  |  |  |
| March |  |  |  |  |  |  |
| April |  |  |  |  |  |  |
| May |  |  |  |  |  |  |
| June |  |  |  |  |  |  |
| July |  |  |  |  |  |  |
| August |  |  |  |  |  |  |
| September |  |  |  |  |  |  |
| October |  |  |  |  |  |  |
| November |  |  |  |  |  |  |
| December |  |  |  |  |  |  |
| **Annual costs** |  |  |  |  |  |  |

**I. In-service training (HIV & ADVIH only) - Include training on ADVIH (categorized with start-up costs)**

Data sources:

- Which employees have attended these trainings?
- Who paid for these courses?
- What are the costs the staff paid: Cost of training, travel, food, hotel, other?

| **Category of personnel** | **Paid by** | **Cost of training** | **Travel** | **Food** | **Hotel** | **Other** | **% allowance** |
| --- | --- | --- | --- | --- | --- | --- | --- |
|  |  |  |  |  |  |  |  |
|  |  |  |  |  |  |  |  |
|  |  |  |  |  |  |  |  |
|  |  |  |  |  |  |  |  |
|  |  |  |  |  |  |  |  |
|  |  |  |  |  |  |  |  |
|  |  |  |  |  |  |  |  |
|  |  |  |  |  |  |  |  |
|  |  |  |  |  |  |  |  |

**J. Waste Management**

Data sources:

- What types of waste are produced by the programme?
- How is waste management financed?
- Is there a waste management company? What are the service fees?
- Identify the unit costs of waste management by?

| **Type of waste (list: ex: incinerator)** | **Paid by** | **Company responsible for waste management** | **Quantity** | **Unit costs** | | **% allowance** |
| --- | --- | --- | --- | --- | --- | --- |
|  |  |  |  | **The end.** | **Econ.** |  |
| Incinerator |  |  |  |  |  |  |
| ADVIH - waste management |  |  |  |  |  |  |
|  |  |  |  |  |  |  |
|  |  |  |  |  |  |  |
|  |  |  |  |  |  |  |
|  |  |  |  |  |  |  |
|  |  |  |  |  |  |  |
|  |  |  |  |  |  |  |
|  |  |  |  |  |  |  |

**Note:** HTS/HIV/AIDS allocation - will depend on site-specific waste management.

**K. Indicators for HIV testing**

Data sources:

| **Months 2019-2020** | **Determine®** | **Unigold®** | **Total HIV+ clients** | **Total HIV- clients** | **Total**  **ADVIH dispensed** | **Total**  **HTS customers** | **Total**  **Clients for all services** | **Comments** |
| --- | --- | --- | --- | --- | --- | --- | --- | --- |
| January |  |  |  |  |  |  |  |  |
| February |  |  |  |  |  |  |  |  |
| March |  |  |  |  |  |  |  |  |
| April |  |  |  |  |  |  |  |  |
| May |  |  |  |  |  |  |  |  |
| June |  |  |  |  |  |  |  |  |
| July |  |  |  |  |  |  |  |  |
| August |  |  |  |  |  |  |  |  |
| September |  |  |  |  |  |  |  |  |
| October |  |  |  |  |  |  |  |  |
| November |  |  |  |  |  |  |  |  |
| December |  |  |  |  |  |  |  |  |
| **Total** |  |  |  |  |  |  |  |  |

**L. Assumptions**

| **Date** | **Hypothesis/Decision** |
| --- | --- |
|  |  |
|  |  |
|  |  |
|  |  |
|  |  |
|  |  |
|  |  |
|  |  |
|  |  |
|  |  |
|  |  |
|  |  |
|  |  |
|  |  |
|  |  |
|  |  |
|  |  |
|  |  |
|  |  |
|  |  |
|  |  |

**Equipment and Consumables - Checklist**

| **Consumables**  Medical and non-medical | **Equipment**  Medical and non-medical |
| --- | --- |
| Cotton | Tables (e.g. wood, plastic; size) |
| Alcoholic wipes | Bench (e.g. wood, plastic; size) |
| HIV testing (Determine; Unigold) | Chairs (e.g. wood, plastic; size) |
| HIV self-tests (ADVIH) | Binders |
| Lancets | Wardrobe |
| Capillary tubes | Bin (e.g. wood, plastic; size) |
| Cotton buds | Fridge (e.g. waist, with freezer) |
| Aprons | Shelves |
| Sterile gloves | PC computer |
| Alcoholic solution | Desktop printer |
| Garbage bag | Examination bed |
| Hydroalcoholic hand solution | Scale for weighing |
| Soap |  |
| Medical waste collectors |  |
| Syringes - 10mls; 5ml; 2ml |  |
| Tapes |  |
| Towels |  |
| A4 paper |  |
| Pen/pencils |  |
| Adhesive tape |  |
| HTS, ADVIH registers |  |

Study of times and movements

Patient information leaflet

of caregivers participating in the study of time and motion

Version 1.0 of 10 April 2019 approved by the WHO Research Ethics Committee (date) and the Ethics Committees of Côte d'Ivoire (opinion, date), Mali (opinion, date) and Senegal (opinion, date).

Hello, I am a…………Solthis and the London School of Hygiene & Tropical Medicine.

ATLAS is a research project that we hope will help us understand how best to introduce HIV self-testing in your country. The study we are conducting is part of the ATLAS project. It is an analysis of the costs of conventional HIV testing, and the costs associated with providing HIV self-testing.

The study of time and motion is intended to estimate the time it takes care providers to provide HIV testing services and HIV self-testing to a patient. This will allow us to estimate the staff costs directly associated with providing HIV care by estimating the time they spend working on this activity.

We are presenting this information to you because we will record the length of your consultation with the health care provider who agreed to participate in this study.

The researcher will be present during your consultation or will stay outside the consultation room according to your preference. If it is convenient for you, we will attend the consultation until the screening test is completed but will leave the room when the test result is read to respect the confidentiality of your clinical appointment. The data is anonymous, your name is not listed.

### More information on the ATLAS programme

If you would like more information about the study, please contact one of the team members. A website dedicated to the ATLAS programme has been opened [(](https://www.google.com/url?q=https://atlas.solthis.org/&sa=D&ust=1552897359532000&usg=AFQjCNFeeDHuOImRkHc0ycsy0zeHGUG_fQ)https://atlas.solthis.org/). You will be able to find there an information leaflet as well as the results of the research at the end of the project.

Study of times and movements - Consent form

The information leaflet, version 1.0 of 20 March 2019, describing the purpose, methods, benefits and risks of research on the costs (individual interviews) of HIV self-testing was read and explained to me. I had the opportunity to have satisfactory answers to all the questions about the study and had enough time to think about my participation.

If you would like more information, please do not hesitate to ask us questions, read the information brochure provided or ask one of our team members to read it to you.

If you agree to participate in the study, we will record your daily activities throughout the day. Please remember that the purpose of this study is not to "control" the quality of care provided, but only to better understand the time it takes to perform HIV tests/self-tests.

Do you have any questions about the study?

Do you agree to participate in the study of time and movement?

If you have no further questions and agree to participate in the study, please sign this form, indicating that I have informed you of your rights as a participant and that you have agreed to participate in the research. Thank you for your time.

PARTICIPANT] I have read the information in the information leaflet for this study (or it was read to me). All my questions about the study and my participation in it have been answered. I freely agree to participate in this study.

_____________________________________________________________________________

Name of participant (in capital letters) Signature Date

_____________________________________________________________________________

Researcher's name (in capital letters) Signature Date

Data collection form - Study of time and movements

| **Study ID:_** |
| --- |

**Date:**

**Researcher:**

**District:**

**Name of the site:**

**Participant ID:_**

| **Model for the delivery of ADVIH** | **Tick** | **Tick more than one box if the health worker works on different models during the observation day and report the type of model in the "Notes" section for each observation.** |
| --- | --- | --- |
| Mobile HIV services team for sex workers |  |  |
| Mobile HIV services team for MSM |  |  |
| Fixed HIV service site for sex workers |  |  |
| Fixed HIV service site for MSM |  |  |
| Mobile HIV services team for DUs |  |  |
| Fixed site of HIV services for DUs |  |  |
| Consult' STI |  |  |
| Sites for index cases |  |  |
| Mobile HIV services team for young people (16-24) |  |  |
| School and University Health Service |  |  |

**Did the study participant sign the consent form?**

|  |  |  |  |  |  | |  |  |
| --- | --- | --- | --- | --- | --- | --- | --- | --- |
|  | 1 | YES |  |  |  |  |  |  |
|  | 2 | NO | ► |  | | Take a break and get the form signed | | |

**Professional level of the study participant:**

| **Professional level** | **Tick** |
| --- | --- |
| Nurse |  |
| Professional HIV counsellor |  |
| Non-professional HIV counsellor |  |
| Volunteer |  |
| Other (specify): |  |

**To be read to the participant :**

**To begin with, I will ask you for some basic information. This will help us to understand how your time is divided between the different services during the day. Please indicate your normal working hours during the week. This includes all the hours you spend at your place of work - including travel time to and from the distribution site if you provide services in communities.**

**Average working hours in the week :**

|  | Start time | | | |  | End time | | | |
| --- | --- | --- | --- | --- | --- | --- | --- | --- | --- |
|  | HH | | MM | |  | HH | | MM | |
| Monday |  |  |  |  |  |  |  |  |  |
|  |  |  |  |  |  |  |  |  |  |
| Tuesday |  |  |  |  |  |  |  |  |  |
|  |  |  |  |  |  |  |  |  |  |
| Wednesday |  |  |  |  |  |  |  |  |  |
|  |  |  |  |  |  |  |  |  |  |
| Thursday |  |  |  |  |  |  |  |  |  |
|  |  |  |  |  |  |  |  |  |  |
| Friday |  |  |  |  |  |  |  |  |  |
|  |  |  |  |  |  |  |  |  |  |
| Saturday |  |  |  |  |  |  |  |  |  |
|  |  |  |  |  |  |  |  |  |  |
| Sunday |  |  |  |  |  |  |  |  |  |
|  |  |  |  |  |  |  |  |  |  |
| Public Holidays |  |  |  |  |  |  |  |  |  |
|  |  |  |  |  |  |  |  |  |  |

| **Notes:** Specify if there are any irregular working hours: |
| --- |

**The following section should be completed by observing one participant at a time for the entire working day. Record each activity observed on a separate line. Use one form per participant. Use the back of the sheet if necessary.**

| **CODE** | **DESCRIPTION OF THE ACTIVITY** |
| --- | --- |
| **ADVIH ADMIN** | Administrative organisational work for the day: decision on the workplace, team preparation time, etc. (if community-based model of care provision) |
| **TRAJET** | Driving time for the dispenser to reach the site, including the time needed to set up the tent (if community care provision model) |
| **TVIH** | Conventional HIV testing session including time spent in pre/post test counselling. (Applies to individual, couple or group counselling) |
| **ADVIH INFO** | Information on HIV (and ADVIH) testing before/without distribution.  Use this code if the customer refuses to test (or self-test) |
| **ADVIH 1** | Primary distribution of ADVIH (may include pre-test counselling, demonstration on how to self-test, waiting for test results and post-test counselling)  (if the distribution is made to a group, note the number of people in "Notes") |
| **ADVIH 2** | Secondary distribution of ADVIH (may include pre-test counselling, demonstration on how to self-test) (if distributing to a group, note the number of people in "Notes") |
| **TVIH/D2** | Conventional HIV testing session including secondary distribution of ADVIH |
| **OTHER SAP** | Other To Patient Services: time allocated to services not directly related to HIV and HIV testing (family planning, PrEP, ART initiation, etc.) provided by the health professional to a client. |
| **NON-SAP** | All the time spent without meeting customers (breaks, waiting, etc.) |


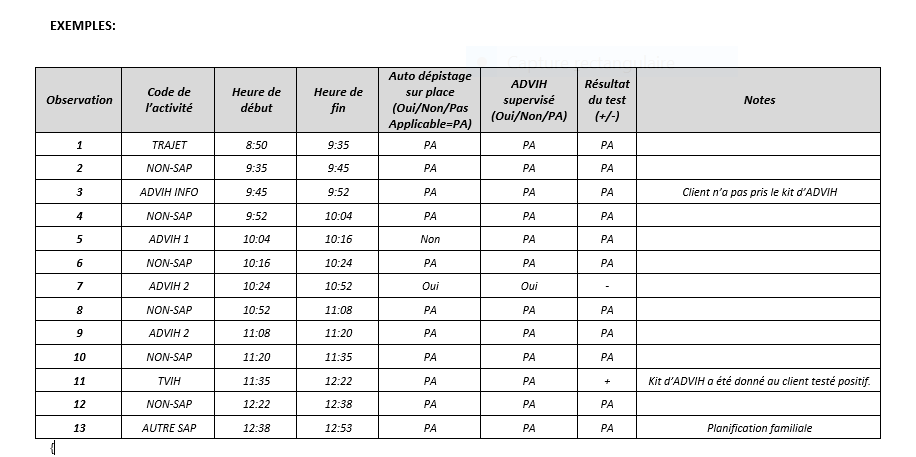


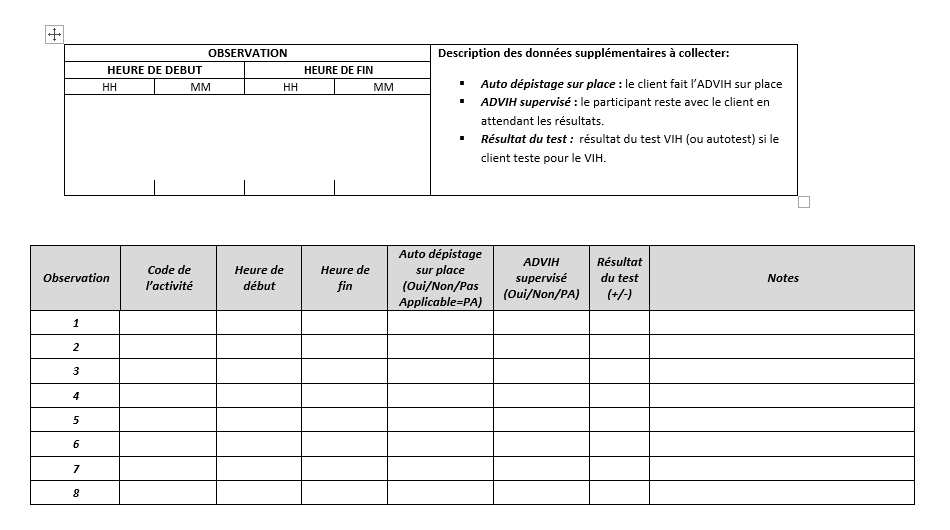


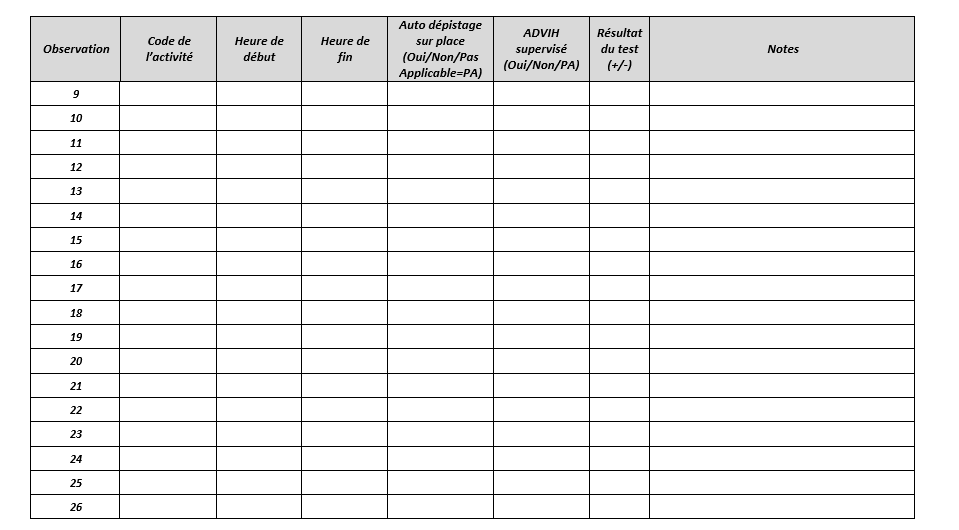


**Ascertaining and managing cases of social prejudice and inciden** **ts**

**ATLAS**

**Reporting and Management of Social Harm and Incidents**

This document is intended to set out the procedures for reporting and processing cases of social harm that may result from the dispensation or use of HIV-positive persons within the framework of the ATLAS project.

The contents of this document apply to and are supported by all the project country teams and all the implementing partners of the ATLAS project. The indications and guidelines mentioned in this document are binding both on the reporting entities (public sites and associations) involved in the provision of HIV and AIDS services and on the Green Lines which receive calls from users.

1. **Reporting of cases of social prejudice**

**What do we mean first of all by social prejudice?**

This includes all **forms of violence** related to the use of HIV:

- **Sexual** including kidnapping by partner or client ;
- **Physical**, being beaten or abused by the partner or client ;
- **Forced** to do the text using force or any other means of blackmail (money, threat of dismissal, etc.);
- **Emotional and psychological,** including isolation, humiliation that can lead to stress or trauma.
- **Verbal abuse** intended to devalue or reject the person;
- **Suicide or attempted suicide that** may result from the discovery of one's status.

**How do we escalate and manage these cases?**

Based on the calls received at the hotline level, the listener, trained to collect the reasons for the calls and provide services relating to ADVIH to the users/callers, records any case of social harm reported by the user/caller on the hotline register.

The listener collects, with the consent of the caller who wishes to share a case, additional data on the circumstances of the incident and the consequences or harm suffered by the caller. Depending on the information thus collected, the listener directs the user to a structure specialising in the type(s) of care appropriate and adapted to the case identified.

This information is systematically notified to the ATLAS project by the Green Line Manager or the Monitoring & Evaluation Focal Point.

Within 48 hours, the ATLAS project manager and his staff carry out an exploratory work in collaboration with the hotline to better describe the incident reported by the listener and ensure that it follows the use of ADVIH.

The Country Project Manager and his/her team will contact the manager(s) of the facility to which the complainant is referred by the hotline to ensure follow-up of the case. The person in charge of this structure informs the ATLAS Project Manager of the result of the diagnosis of his services and the nature of the care that the person is supposed to receive.

The person in charge of the care structure provides the ATLAS Project Manager, by e-mail, with evidence of the actual care of the person referred and received by his services.

The country project leader acknowledges receipt of the documents by e-mail and checks all the evidence shared by the structure manager and sends a notification of closure of the incident to the Green Line manager within 48 hours.

If the Project Manager finds a breach in the services provided, a notification is sent to the person in charge of the structure. The notification specifies the elements deemed to be missing from the care so that the facility manager can ask his services to bring the person back to ensure that the care is complete.

The newly produced evidence is shared with the Project Manager who acknowledges receipt and carries out the verification before sending, if necessary, a notification of closure of the incident to the Green Line within 48 hours.

1. **Misuse of ADVIH**

Acquisition of ADVIH by purchase or exchange in kind

In the "incident" column of the hotline register, the listener informs about all the cases of acquisition of ADVIH not free of charge reported by users/callers.

With the caller's informed consent, the caller collects additional information on the circumstances of receipt of the ADVIH by sale or exchange in kind which he or she immediately transmits to the Green Line Manager or the M&E Focal Point.

These data are automatically forwarded to the Country Project Manager who, within 48 hours, confidentially contacts the person in charge of the structure or association from which the PLWHA(s) in question originated.

The Project Manager's contact with the person in charge of the association or ADVIH reception structure is notified by email to the Project Director.

An investigation is opened discreetly and is conducted jointly between the head of the structure or its mandate and the Project Manager or his mandate.

After fifteen days of collecting and cross-checking information on the circumstances of the sale or exchange of ADVIH, a detailed report setting out the responsibilities is produced and sent to the ATLAS Project Director.

After consultation with the project coordination team and the Solthis COPIL, the Project Director informs Unitaid within 48 hours for advice and action to be taken on the incident.

The decision taken by Solthis and ATLAS following Unitaid's recommendations will be notified to the structure by official letter. The incident will thus be closed in the absence of the opening of legal proceedings.

In the event of legal action, the deliberation of the competent court in the matter will de facto result in the incident being closed. A notification will be sent to the Green Line to notify it of the closure of the incident.

All documentation produced will be filed and archived until the end of the project.

**Data Management Plan**

**ATLAS**

**Data Management Plan (DMP)**

*version of 5 August 2019*

*This data management plan is publicly available on https://dmp.opidor.fr/.*

*It follows the plan recommended by the Digital Curation Centre (DCC - http://www.dcc.ac.uk/).*

# 1. Data collection

## **1.1 What data will you collect or create?**

The different research strands and the collections associated with each of them are detailed in the project protocol, which can be consulted on [https://atlas.solthis.org/.](https://atlas.solthis.org/)

The nature of the data collected is of four types : (i) qualitative survey data (audio recordings or note-taking of individual and group interviews, face-to-face or by telephone); (ii) quantitative telephone survey data (voucher survey); (iii) time and movement survey data; (iv) financial data (financial reports, specific collection from provider sites).

## **1.2 How will the data be collected or created?**

### *Qualitative survey data*

These are individual and group interviews. The interviews will be recorded (audio, MP3 format or equivalent) with the consent of the participants. For individual interviews, if the respondent refuses the audio recording, the rice interviewer will take manual notes.

Each interview will be numbered according to the survey or sub-survey to which it corresponds and the country of collection. The interview number will be recorded on the consent form.

The interviews will be conducted by senior anthropologists with the support of research assistants trained in social sciences and interviewing. The qualitative team will meet at regular intervals to coordinate field activities and to check the quality of the interviews.

Audio recordings and note-taking will be transcribed (text file with formatting) in French (with translation if necessary for interviews conducted in another language). During the transcription, the interviews will be coded (change of surnames and first names for example) in order to pseudonymise them.

### *Data from quantitative telephone surveys*

The telephone survey (WP Enquête Coupons) will be carried out by Ipsos Côte d'Ivoire, which has a CATI (Computer Assisted Telephone Interviewing) platform in Abidjan. Three toll-free numbers will be set up (Ivory Coast, Mali, Senegal). All calls will be routed to the CATI platform via gateways set up in each country (voice over IP). During the call reception phases (phase 1 of the survey), incoming calls will be automatically directed to an investigator from Côte d'Ivoire, Mali or Senegal depending on the origin of the call. Prior to the start of collection, interviewers will have undergone specific training on the survey and its questionnaire, conducted by both Ipsos Côte d'Ivoire supervisors and the research team.

The PACCI programme, an Ivorian research institution based in Abidjan and partner in the ATLAS project, will develop a platform dedicated to data capture, telephone appointment management, production of survey monitoring dashboards and export of collected data in a text-based format (CSV).

Analytical files dedicated to scientific analysis will be generated from this collection platform. These files will be generated in an open format (CSV type text files). These analytical files of the coupon survey will not contain any personal data.

Quality control of data collection will be ensured through the implementation of consistency checks at the time of data entry, double-taping by supervisors of interviews conducted by interviewers and checks on the analytical basis during the course of the survey. As the voucher survey will be organised in 3 waves, an in-depth analysis of the data from the first wave will be carried out before waves 2 and 3 are conducted, and operational procedures will be adjusted accordingly.

### *Data from the Time and Movement Survey*

This survey will be conducted by two junior economists supervised by two senior economists. Field observations and the completion of the questionnaires will be carried out by the two junior economists on paper. The data collected will then be entered by the two senior economists in Excel format.

The team of economists will meet at regular intervals to check the smooth running of the surveys and the quality of the data collected.

### *Financial data*

The same team of economists will go to collect financial data from a sample of health and community structures involved in the distribution of HIV self-testing in the ATLAS programme. The data collected will also be in Excel format.

# 2. Documentation and Metadata

## **2.1 What documentation and metadata will be associated with the data?**

### *Qualitative survey data*

Each interview transcript will be accompanied by an interview report including: the context in which the interview took place, socio-demographic characteristics and a presentation of the interviewee(s) and a summary.

### *Data from quantitative telephone surveys*

The analytical files generated from the collection platform will be documented according to the DDI (*Data Documentation Initiative* [http://www.ddialliance.org/)](http://www.ddialliance.org/) specification dedicated to the documentation of quantitative survey data in the humanities and social sciences. The documentation of the variables will be carried out using the *Nesstar Publisher* software from the *Norwegian Centre for Research Data*. This software allows, in particular, the production of a detailed dictionary of variables and the export of labelled data files in Stata and SPSS format. A copy of the data in CSV format (text files) will be systematically kept.

### *Data from the Time and Movement Survey*

The time and motion survey files will also be documented according to the DDI specification.

### *Financial data*

The collection will be based both on the various reporting documents produced by the structure generating the dispensing site (financial reports, activity reports, etc.) and on a grid currently being developed by the team of economists.

# 3. Ethical and legal aspects

## **3.1 How will you manage the ethical aspects?**

The research protocol was evaluated and authorised by 4 ethics committees:

- OMS : Ethical Research Committee, date :
- Côte d'Ivoire: National Ethics Committee for Life Sciences and Health, date: 28 May 2019
- Mali: Ethics Committee of the Faculty of Medicine and Pharmacy of the University of Bamako, date:
- Senegal: National Ethics Committee for Health Research, date: 26 July 2019

All the consent forms and information notices are available in the annex to the protocol, on the website [https://atlas.solthis.org/.](https://atlas.solthis.org/)

The consent forms will be kept for 5 years after the end of the project (i.e. until the end of 2026) in a locked cupboard in the Solthis premises located in Abidjan for the consent forms collected in Côte d'Ivoire, Bamako for the forms collected in Mali and Dakar for the forms collected in Senegal.

### *Qualitative survey data*

Prior to any face-to-face interview, an informed consent form will be signed by the respondent. Specific consent will be sought for audio recordings. In order to enable respondents to exercise their right of access, rectification and opposition, the unique number of the interview will be recorded on the consent form given to the respondent.

For interviews conducted by telephone, consent will be sought verbally from the respondent and his/her anonymity will be preserved. A verbal consent form will be completed by the rice interviewer. The respondent's contact data will be destroyed immediately after the telephone interview. The interview number will be given to the respondent and it will be explained to him/her that he/she must keep it if he/she wishes to be able to exercise his/her right of access, rectification and/or opposition at a later date.

The audio recordings of the interviews will be kept for the time of their transcription and pseudonymisation. Each researcher undertakes to destroy the audio recordings by the end of the project at the latest (end 2021).

As mentioned in the information sheets, the pseudonymised data will be kept for a maximum of 5 years after the end of the project, i.e. until the end of 2026 at the latest. Only anonymised data will be subject to long-term archiving (see chapter "Selection and preservation").

### *Data from quantitative telephone surveys*

Given the nature of the survey (anonymous calls on a free telephone line), consent to participate in the study will be sought orally. The collection of consent will be notified and time-stamped in the database.

The data collected will be anonymous by nature, unless the respondent provides one or more telephone numbers in the following cases: (i) wishes to be called back at a later date to continue the interview; (ii) is eligible for phase 2 of the survey (additional questionnaires by callback three months later), agrees to be recontacted and to leave a contact number; (iii) agrees to be recontacted for a complementary qualitative interview and to leave a contact number. In the absence of a telephone number, the granularity of the data collected will not allow the identification of individuals by cross-checking the information. The telephone numbers collected will be stored in a specific table in the database and deleted at the end of the survey (by the end of 2021 at the latest).

If the respondent has given a telephone number, he or she will be able to exercise his or her right of access, rectification and/or opposition (identification possible via the telephone number) during the duration of the study (2019-2021). If the respondent has not provided the interviewer with a telephone number, the exercise of the right of access, rectification and/or opposition will no longer be possible once the interview has been completed.

### *Data from the Time and Movement Survey*

Informed and written consent will be sought from the delivery agents selected for this study. A participation number will be mentioned on the consent form to enable them to exercise their right of access, rectification and/or opposition.

As mentioned in the information sheets, the raw data will be kept for a maximum of 5 years after the end of the project, i.e. until the end of 2026 at the latest. Only data that have been anonymised will be archived for a long period of time (see chapter "Selection and preservation").

### *Financial data*

All hard copies of the recordings will be kept by the study teams in locked cabinets. Electronic copies of the data will be stored in password-protected or restricted access computers on Solthis' Microsoft SharePoint server. Access to the records will be restricted to economist teams and ATLAS team members as required.

The raw data collected will be kept for a maximum of 5 years after the end of the project, i.e. until the end of 2026 at the latest. Only data that has been subject to specific processing to avoid identifying the structures surveyed (aggregated data for example) will be archived for a long period (see the chapter "Selection and preservation").

## **3.2 How will you take into account copyright and intellectual property aspects?**

The intellectual property rights to the project data and their derivatives remain with the various parties that created or produced the data. However, the different members of the Atlas consortium have committed to share the data produced among themselves.

Any publication or scientific communication carried out within the framework of the ATLAS project and relating to project data must be discussed upstream by the project's scientific steering committee and must be part of the project's valorisation plan.

Anonymised research data (see section 5. Selection and Preservation) that will be archived in an open access archive will be distributed under a *Creative Commons Attribution-Share Alike 4.0 International* License [(](https://creativecommons.org/licenses/by-sa/4.0/)https://creativecommons.org/licenses/by-sa/4.0/).

The research institutions of the project have furthermore all contractually agreed to provide a non-exclusive, irrevocable, worldwide, royalty-free, sub-licence to WHO on behalf of UNITAID to use the anonymised data (see section 5. Selection and Preservation) of the project and the scientific publications produced for non-commercial public health, education and research purposes.

# 4. Storage and backup

## **4.1 How will the data be stored and backed up during the research project?**

### *Qualitative survey data*

The audio recordings of the interviews will be stored on the personal computer of the researcher conducting the interview and will be password-protected.

The transcribed and pseudonymised interviews will be deposited on the <https://cloud.atlasti.com/>platform dedicated to the collaborative analysis of qualitative data and in particular to the thematic codification of interviews. This platform is a product developed and marketed by *Scientific Software Development GmbH, which* specialises in software for scientific qualitative analysis. The platform is secure and the access is individual and private. According to the service's privacy policy [(](https://atlasti.com/privacy/)https://atlasti.com/privacy/), the "ATLAS.ti Cloud" platform complies with the General Data Protection Regulation (GDPR) of the European Union. Communications will be encrypted (https), individual access (user account) with rights management.

### *Data from quantitative telephone surveys*

The data collection platform will be hosted on a secure computer server located on the premises of the PACCI programme. Access to the platform (investigators, supervisors, researchers) will only be possible through an encrypted connection (https) and an individual user account (the rights of each actor can be defined separately).

Statistical analyses will be carried out using anonymised analytical files extracted from this platform. The analyses will be carried out with the R software and hosted on the infrastructure of the TGIR Huma-Num [(https://www.huma-num.fr/)](https://www.huma-num.fr/) which provides an R Studio Pro server [(https://r-tools.huma-num.fr)](https://r-tools.huma-num.fr) and a Git version manager ([https://gitlab.huma-num.fr)](https://gitlab.huma-num.fr) located on secure servers at the Centre national de la recherche scientifique (CNRS) in France. Communications are encrypted (https), with individual access and rights management per user.

### *Data from the Time and Movement Survey*

The Excel files will be hosted on the Microsoft SharePoint platform of the NGO Solthis (Solthis is the carrier of the ATLAS programme). Access to the files will be limited to members of the research team involved in the economic analysis. Communications are encrypted (https) and individual rights (user accounts).

Statistical analyses will be carried out on the TGIR Hum-Num platform (see previous point).

### *Financial data*

The data collected will also be in Excel format, and hosted on Solthis' Microsoft SharePoint platform.

## **4.2 How will you manage access and security?**

All the computer servers used (internal servers or services provided by third parties) are secure. All communications are encrypted and access rights are systematically managed individually (see previous section).

# 5. Selection and preservation

## **5.1 What data must be kept, shared and/or preserved?**

Personal data (nominative data or data allowing the identification of individuals by cross-checking information) will be kept for a maximum of 5 years after the end of the project, i.e. until the end of 2026 at the most. Similarly, the consent forms will be kept for the same period in order to allow the persons surveyed to exercise their right of access, rectification and/or opposition (see section 3.1).

Only anonymised data generated from the raw data will be archived.

### *Qualitative survey data*

A procedure for anonymising the pseudonymised transcripts of the interviews will be put in place. The interview reports will be taken up one by one and all passages containing information that could identify the interviewee or those mentioned by the interviewee will be deleted (with an indication of the deletion). In case of doubt, deletion will be encouraged. Anonymised interview reports will be validated collectively by the qualitative research teams.

### *Data from quantitative telephone surveys*

Anonymisation of the data will be carried out when generating analytical data files from the data collection platform.

### *Data from the Time and Movement Survey*

Anonymised analytical files will be produced at the end of the project. As the survey was conducted among a specific population (delivery agents) of small size, data anonymization tools such as *ARX Data Anonymization Tool* [(https://arx.deidentifier.org/)](https://arx.deidentifier.org/) will be used to guide the selection and recoding of variables.

### *Financial data*

In a similar way, anonymised analytical files (which do not allow the dispensing sites to be identified) will be generated. If necessary, the use of aggregated data will be considered.

## **5.2 What is the long-term preservation plan for the datasets?**

Anonymised data files will be deposited on the Zenodo data repository: https://www.zenodo.org/communities/atlas-research/. Zenodo is a free and public repository, hosted by CERN and supported by the European Union.

# Sharing of data

## **6.1 How will you share data?**

The data deposited on Zenodo will each have a DOI (Digital Object Identifier) allowing their permanent identification over time. These DOIs will notably be used in scientific publications generated from these data.

## **6.2 Are there any restrictions on data sharing?**

For the duration of the project, the data files will be made available on demand, while the research teams publish the results of their analyses.

Once the project is completed and the corresponding articles published, the anonymised quantitative data files will be freely available and distributed under a *Creative Commons Attribution-Share Alike 4.0 International* License [(](https://creativecommons.org/licenses/by-sa/4.0/)https://creativecommons.org/licenses/by-sa/4.0/).

Concerning qualitative data, considering that the anonymisation process described above could be a source of error and that the identification of some of the people surveyed could still be theoretically possible by cross-referencing information, access to the archived data will be restricted. Access, via the Zenodo platform, will be made on request, by academic teams and will be subject to the signature of a confidentiality agreement.

# Responsibilities and Resources

## **7.1 Who will be responsible for data management?**

Each work package is coordinated by a scientific manager (see protocol) who is responsible for ensuring that the activities of the work package are carried out correctly.

The principal investigator will ensure the overall implementation of the data management plan.

A detailed organisation chart of roles and responsibilities will be developed at the beginning of the project.

## **7.2 What resources will you need to implement your plan?**

The human, material and software resources required for the implementation of the data management plan have been foreseen and included in the project budget.

**Summary of the project document validated by Unitaid**

*November 2018*
